# The challenges of direct Claisen–Schmidt condensation in the synthesis of polyhydroxylated bischalcones: experimental results supported by DFT calculations

**DOI:** 10.3762/bjoc.22.114

**Published:** 2026-10-12

**Authors:** Rui Pereira, Mariela M Nolasco, Vera L M Silva, Eduarda Fernandes

**Affiliations:** 1 LAQV-REQUIMTE, Laboratory of Applied Chemistry, Department of Chemical Sciences, Faculty of Pharmacy, University of Porto, 4050-313 Porto, Portugal; 2 CICECO-Aveiro Institute of Materials, University of Aveiro, 3810-193 Aveiro, Portugal; 3 LAQV-REQUIMTE and Department of Chemistry, University of Aveiro, 3810-193 Aveiro, Portugal

**Keywords:** bisacetophenones, bischalcones, Claisen–Schmidt condensation, density functional calculations, Fukui functions, green chemistry

## Abstract

The synthesis of chalcones commonly relies on the Claisen–Schmidt condensation, an attractive reaction owing to its operational simplicity and favorable atom economy. However, its application to acetophenones bearing multiple hydroxy substituents can be problematic, often requiring hydroxy protection and subsequent deprotection, thereby diminishing the sustainability advantages of the approach. In this work, density functional theory (DFT) calculations were performed at the CAM-B3LYP/6-311++G(d,p) level to provide a qualitative electronic rationale for the factors that may account for this limitation. The nucleophilic behavior of the starting species was examined under basic, acidic, and metal-assisted conditions, using molecular models both with and without the hydroxy groups to evaluate the hypothesis that competing nucleophilic sites could interfere with the desired condensation and hinder the reaction. The results indicated that strong basic conditions favor extensive phenol deprotonation, generating several competing nucleophiles. In this situation, the resulting phenolate species were more nucleophilic than the corresponding enolates, favoring undesired reaction pathways. Coordination of lithium or calcium to the two phenolate groups decreased their nucleophilicity but failed to suppress the unwanted reactivity of the remaining phenolate. In contrast, under acidic conditions, the enol species exhibited greater nucleophilic character than the phenolic groups, providing no support for the proposed competition between phenolates and enolates as the sole explanation for the unsuccessful reaction. Nevertheless, the acidic environment may facilitate isomerization of the bischalcone products, which could subsequently promote degradation and/or undesired cyclization processes. Taken together, experimental and computational results indicate that the failure of the reaction arises from multiple competing factors rather than from a single mechanistic limitation. Among the conditions investigated, the acid-mediated pathway appears to be the most promising starting point for further optimization of the synthesis of polyhydroxylated bischalcones.

## Introduction

The Claisen–Schmidt condensation has been the backbone of chalcones’ (1,3-diarylprop-2-en-1-ones) synthesis for over 100 years [1], following the first report by Claisen in 1881 [2]. This method is a specific case of cross-aldol condensation, in which two different carbonyl-based molecules are mixed, one capable of enolization to act as a nucleophile and the other incapable of the same feat, to act as an acceptor [3–5]. It remains one of the primary methods in organic synthesis for carbon–carbon bond formation, owing to its broad substrate scope and mild reaction conditions, despite its below-average reaction rate and susceptibility to side reactions [6]. Cross-aldol condensation is also a very efficient reaction regarding atom economy and lack of hazardous and toxic reagents, making it an optimal approach in line with green chemistry principles [6–8].

This reaction is based on the chemistry of the carbonyl group, specifically ketones and aldehydes. Keto–enol tautomerism allows these molecules to act as nucleophiles or electrophiles, depending on the environment and catalysts involved [9]. With the right combination of two molecules and the right catalyst, new carbon–carbon bonds form, creating extended molecules. However, the efficiency of this reaction is greatly reduced when using molecules with multiple nucleophilic groups, such as polyhydroxylated compounds. It becomes difficult to control which nucleophile reacts first and where it will react, and many side reactions can occur. In these cases, both the yield and the ease of purification of the reaction products drop considerably.

Due to this obstacle, most reports in the literature avoid the reactivity of these additional nucleophilic groups by using protecting groups to render them temporarily inert and then carrying out the intended cross-aldol condensation [10–13]. Only the added protecting groups are then removed. This strategy has been successful for many decades, allowing chemists to prepare many derivatives that would otherwise be impossible to synthesize. Yet, this adaptation of the synthetic pathway contradicts the initial advantages described for the cross-aldol condensation. The addition of protecting- or deprotecting-group steps severely reduces atom economy and introduces many hazardous and toxic reagents into the synthesis, necessary to introduce or remove these protecting groups [14–15]. Therefore, some authors have devoted their research to finding ways to perform cross-aldol condensation with multiple nucleophilic groups, without the need for protecting groups [16–18].

Chalcones (or 1,3-diarylprop-2-en-1-ones) belong to the flavonoid family and are a major class of natural products commonly found in plants, fruits, vegetables, tea, and other foodstuffs. They are also well recognized for their pharmacological activities [19–20]. The importance of the present research becomes particularly clear for novel chalcone derivatives, especially bischalcones. Bischalcones are a subclass of chalcones with growing interest from a photophysical and medicinal point of view, and thus, efforts to improve their synthesis have been made [21–23]. This is necessary because the efficiency of the cross-aldol condensation with these molecules is even more hindered by the presence of other nucleophiles, and the protection and deprotection reactions contribute to the production of increased amounts of waste and a decrease in yield [21].

Some authors have been successful in carrying out cross-aldol condensation under classic basic or acidic conditions of substrates with a reduced number of hydroxy groups, either one or two, usually hydroxy groups adjacent to carbonyl groups [24]. Other authors use metal-based basic salts to coordinate with the substrates to accelerate the reaction [16–17].

In this context, the initial objective of this work was to establish a successful method for the cross-aldol condensation of a polyhydroxylated bisacetophenone with 4-methoxybenzaldehyde. However, none of the methods and reaction conditions investigated afforded the expected product. Therefore, the subsequent objective was to investigate this reaction and evaluate the hypotheses proposed to explain the experimental observations using DFT calculations. In this study, DFT calculations were employed as qualitative tools to interpret chemical reactivity, providing an electronic-structure rationale for the experimentally observed behaviour and identifying the molecular sites most susceptible to nucleophilic and electrophilic attack. This approach was intended to provide insight into the factors underlying the failure of the tested reaction conditions to yield the desired product.

## Experimental

### Synthesis

Compound **1**, 1,1'-(2,4,6-trihydroxy-1,3-phenylene)bis(ethan-1-one), was obtained as a white solid in 80% yield, with a melting point of 161.6–163.4 °C. The compound was prepared following a procedure previously described by our group [22–23]. The characterization data were in agreement with the data previously reported by our group [22–23] and with the literature values (mp 143–175 °C [25] and 168–175 °C [26]).

The experimental protocols of all the attempted methods for the cross-aldol condensation of 1,1'-(2,4,6-trihydroxy-1,3-phenylene)bis(ethan-1-one) (**1**) with 4-methoxybenzaldehyde, under basic, acid, and metal-assisted conditions (as described in Table 1 of the Results and Discussion section) are provided in Supporting Information File 1.

### Computational details

To study the reactivity of the initial molecules under different reaction conditions, density functional theory (DFT) calculations were used to assess molecular stability and reactivity descriptors for the molecule in question [27–29]. CAM-B3LYP with 6-311++G(d,p) basis set was the functional used. B3LYP is a generalized hybrid-gradient functional that incorporates a 20% mixture of exact exchange with additional semiempirical adjustments to both semi-local exchange and correlation components [30]. CAM-B3LYP (Coulomb-attenuating method) is an optimization of the B3LYP functional for better predictions of long-range interactions and intramolecular charge transfer [30]. All structures were initially optimized using the CAM-B3LYP functional with 6-311++G(d,p) basis set in the gas phase. A re-optimization was conducted at the same functional and basis set level employing the implicit SMD solvation model with methanol as the chosen solvent [31]. All optimized structures were free of any imaginary frequencies, proving the minimum energy state for the system. In specific cases, a quadratically convergent SCF procedure (SCF=QC) was needed to reach a minimum of energy. Additional information concerning the electronic structures of the optimized geometries was obtained by a natural bond orbital (NBO) analysis [32]. All calculations were performed with the Gaussian 09 program (G09), version A.02 [33], and visualized with GaussView, version 6 [34].

The reactivity parameters were calculated using the condensed Fukui functions for every individual atom (k) [35–38]. By using particular population analysis, it is possible to discover the number of electrons (*N*) associated with each atom, according to Equations 1–3.


[1] $$f_{\text{k}}^{+}=q_{\text{k}}(N_{0}+1)-q_{\text{k}}(N_{0})$$

[2] $$f_{\text{k}}^{-}=q_{\text{k}}(N_{0})-q_{\text{k}}(N_{0}-1)$$

[3] $$\Delta f(r)=|f_{\text{k}}^{+}-f_{\text{k}}^{-}|$$

Where 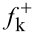 stands for the electrophilicity of a local site and is measured by the electron density (*q*) following the addition of an electron (*N* + 1) to the system, and 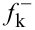, nucleophilicity of a local site, is measured after the removal of an electron from the system (*N −* 1). Δ*f*(*k*) is the dual descriptor, and it defines the affinity for nucleophilic or electrophilic attack. If Δ*f*(*r*) > 0, the site is electrophilic, but if the value is <0, the atom tends to be more nucleophilic; lower numbers indicate increased nucleophilicity [35,39].

To calculate the deprotonation rates of the molecule, a thermodynamic cycle was considered for each deprotonation state, in which a molecule of a reference conjugate base receives a proton from the target molecule, yielding a reference acid (with an experimentally known p*K*_a_) and the deprotonated compound under study [40–42]. This approach, known as the proton exchange method, is considered more reliable because the number of charged species is conserved on both sides of Equation 4, thereby allowing cancellation of some of the errors incurred in a continuum solvent calculation. The calculated p*K*_a_ values should be interpreted as qualitative/semi-quantitative indicators of relative acidity. Phenol was used as the reference molecule for its chemical proximity to the experimental molecules, with an experimental p*K*_a_ of 14.33 [42].


[4]

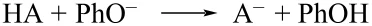



**Scheme 1:**
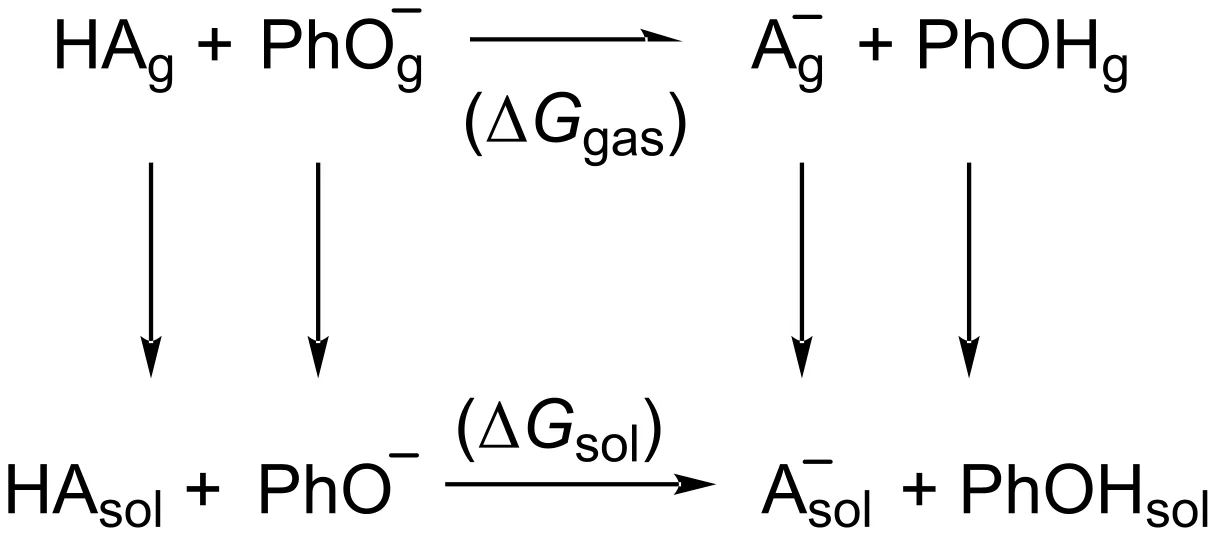
Thermodynamic cycle of deprotonation in gas phase and in methanol solution with phenol as the reference acid.

To compute the free energy difference for this deprotonation reaction in the solvation model (Δ*G*_sol_), the free energy difference of the reaction in the gas model is added to the solvation energy component of each reagent and product, as shown in Scheme 1 [42].

The respective equations used for the calculation of the relative p*K*_a_’s (Equations 5–8) are as follows [42].


[5] $$\text{p}K_{\text{a}}(\text{HA})=\frac{\Delta G_{\text{sol}}^{\text{*}}}{RT\ln(10)}+\text{p}K_{\text{a}}(\text{Href})$$

[6] $$\Delta G_{\text{sol}}^{\text{*}}=\Delta G_{\text{g}}^{*}+\Delta \Delta G_{\text{sol}}^{\text{*}}$$

[7] $$\Delta G_{\text{g}}=G_{\text{gas}}({\text{A}}^{-})+G_{\text{gas}}(\text{HRef})-G_{\text{gas}}(\text{AH})+G_{\text{gas}}({\text{Ref}}^{-})$$

[8] $$\begin{array}{c} \Delta \Delta G_{\text{sol}}^{\text{*}}=(G_{\text{sol}}({\text{A}}^{-})-G_{\text{gas}}({\text{A}}^{-}))+(G_{\text{sol}}(\text{PhOH})-\\ G_{\text{gas}}(\text{PhOH}))-(G_{\text{sol}}(\text{HA})-G_{\text{gas}}(\text{HA}))-\\ G_{\text{sol}}({\text{PhO}}^{-})-G_{\text{gas}}({\text{PhO}}^{-})) \end{array}$$

The binding energy for the metal complexes was calculated using the formula represented in Equation 9:


[9] $$E_{\text{binding}}=E_{\text{monomer}+\text{M}}-(E_{\text{monomer}}+E_{\text{M}})$$

where *E*_monomer+M_ is the energy of the complex of metal ion(s) coordinated with monomer, *E*_monomer_ and *E*_M_ are the energy of monomer and metal ions, respectively.

## Results and Discussion

**Scheme 2:**
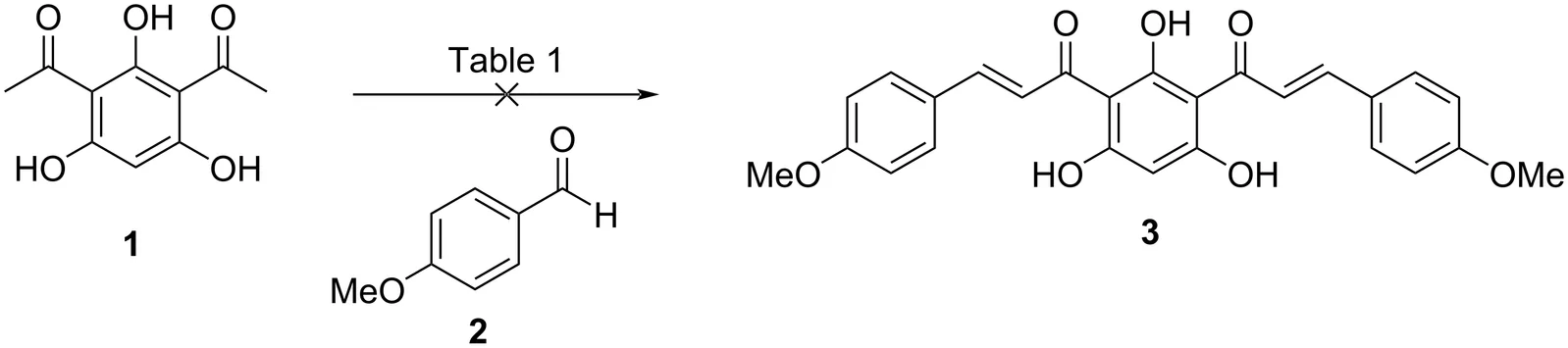
Attempted syntheses of bischalcone **3**.

In this work, the direct route for the synthesis of bischalcone **3** was the cross-aldol condensation of 1,1'-(2,4,6-trihydroxy-1,3-phenylene)bis(ethan-1-one) (**1**) with 4-methoxybenzaldhyde (**2**), represented in Scheme 2. The different reaction conditions tested are described in Table 1.

**Table 1:** Reaction conditions tested for the synthesis of bischalcone **3** (Scheme 2).

reaction	catalyst^a^	solvent	temperature

i)	KOH (20%)	MeOH	rt
ii)	CaCl_2_/KOH	MeOH	65 °C
iii)	LiHMDS	THF	50 °C
iv)	LiOH	MeOH	rt
v)	CaO	MeOH	50 °C
vi)	BF_3_·Et_2_O	dioxane	rt
vii)	BF_3_·Et_2_O	dioxane	80 °C
viii)	Amberlite 15	MeOH	rt (ultrasound)
ix)	*p*-TSA (cat)	MeOH	rt

^a^Along the article, whenever the catalyst was used in stoichiometric quantities or in excess, we refer to the reaction as an acid-/ base-promoted condensation or metal-assisted condensation, instead of acid-/base-/metal-catalyzed condensation.

All methodologies were tested at least twice but failed to yield the desired bischalcone. Base-promoted condensation (KOH, CaCl_2_/KOH, LiHMDS, and LiOH) (Table 1, reactions i–iv) resulted in the complete consumption of the starting reactant **1**, yet no product was detected, only signs of degradation. Using CaO (Table 1, reaction v) led to full recovery of both reactants. The application of Ca- and Li-based bases aimed to limit the reactivity of the initial molecule via metal complexation. While some literature reports describe successful direct cross-aldol condensation between hydroxy ketones and aldehydes using this strategy, all attempts in this case were unsuccessful.

Acid-promoted condensation with BF_3_·Et_2_O (Table 1, reactions vi and vii) induced a color change in the reaction mixture, but both starting reactants remained mostly intact, with a greater difference observed at higher temperatures. Reactions in the presence of a solid-supported acid such as Amberlite 15, assisted by ultrasound (Table 1, reaction viii), failed to produce any product, and both reactants were recovered. Reaction with a catalytic amount of *p*-toluenesulfonic acid (*p*-TSA) (Table 1, reaction ix) resulted in the complete depletion of compound **1** and the formation of an orange spot at the bottom of the TLC plate, regardless of the eluent system used. Comparison with reference compound **3** showed no presence of it in the reaction mixture. No identifiable product was obtained from the crude reaction mixture, and isolation attempts failed.

### Chemical reactivity exploration

Upon verifying that the reaction failed in all the reaction conditions tested, a hypothesis was proposed. The reaction fails because the nucleophilicity of the required atoms for the Claisen–Schmidt condensation is inferior to the nucleophilicity of the remaining hydroxy groups. To test this hypothesis, the chemical reactivity of the optimized initial nucleophile was investigated using Fukui functions calculated with natural bond orbital (NBO) charges, achieved with the Gaussian 09 program (G09), version A.02 [33,43]. These were calculated for each atom through DFT calculations at the CAM-B3LYP/6-311++G(d,p) basis set level of theory within an implicit SMD solvent model for methanol (see Supporting Information File 1). Methanol was the only solvent used for uniformization purposes and to allow comparability between the different nucleophilicities within the same environment.

This study is structured around three main approaches: base-promoted, acid-promoted, and metal-assisted base-promoted condensation. Each approach is first examined using a model reaction with a substrate lacking hydroxy groups, followed by analysis of molecule **1** (Scheme 2) using the same methodology. This hypothesis will be tested in all these reaction conditions by comparing the results with and without additional hydroxy groups.

### Base-promoted condensation

**Model reaction:** This study began with the analysis of the cross-aldol condensation between a model bisketone and an unsubstituted aromatic aldehyde in methanol. The reaction was first conducted under basic conditions, following the method described by L. Claisen and Claparède [2]. The key difference from the classic condensation is the presence of two ketone moieties within the same structure, allowing the reaction to occur twice. The model bisketone was dissolved in methanol, a protic solvent, and potassium hydroxide was added to achieve a concentration of approximately 1.8 M KOH. Methanol was chosen as the solvent because the reagents have low solubility in the aqueous mixture. In solution, hydroxide ions will produce potassium methanolate in an equilibrium reaction (Equation 10), as shown below, which will be the dominant base in solution [44]. The conversion rate of this reaction depends on the ratio of methanol to KOH, but it is always close to full conversion in dilute solutions, so the concentration of KOH added will be similar to the final concentration of potassium methanolate generated [44–45].


[10]

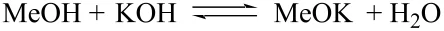



**Scheme 3:**
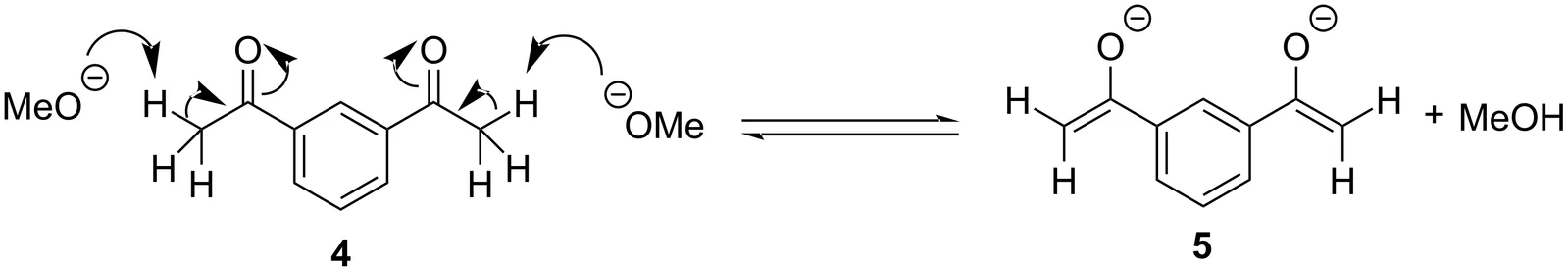
Enolate **5** formation step in a basic medium.

The methanolate (MeO^−^), will remove the acidic protons of bisketone **4,** leading to the formation of a double enolate **5** (Scheme 3) [45].

**Figure 1:**
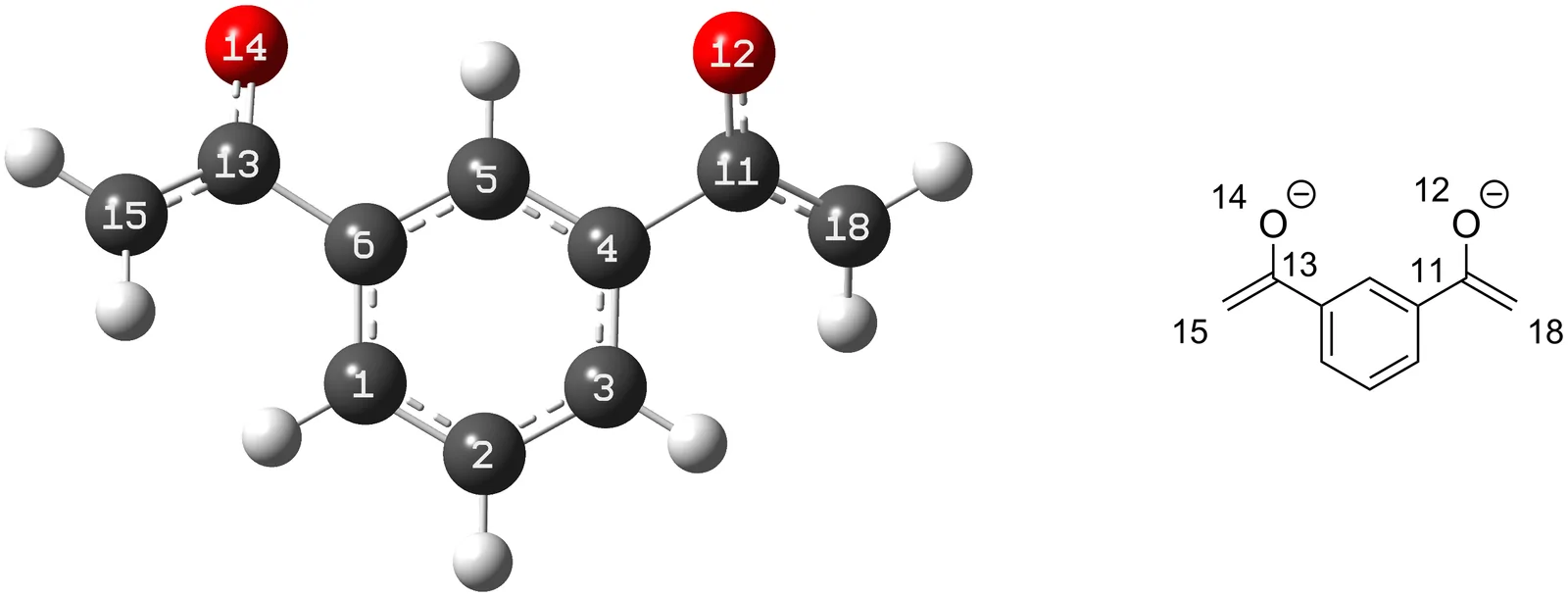
Optimized structure of **5** in methanol phase, at the CAM-B3LYP/6-311++G(d,p) basis set level.

The resulting molecule, unlike a common enolate, has two nucleophilic sites within the same structure, reducing the nucleophilicity of each site. This aspect has implications for the reactivity of each site, as shown in Table 2, acquired from the condensed Fukui function values (as described in the Computational details section) obtained from NBO analysis performed with the optimized structure of **5** at the CAM-B3LYP/6-311++G(d,p) basis set level (Figure 1).

**Table 2:** Condensed Fukui function values obtained for compound **5** from NBO charges in methanol solution (SMD solvent model) at the CAM-B3LYP/6-311++G(d,p) basis set level.

atom	type	*q_k_*(*N*)	*q_k_*(*N* – 1)	*q_k_*(*N* + 1)	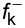	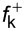	Δ*f*(*r*)

11	C	5.66283	5.65255	5.67577	0.01028	0.01294	0.00266
12	O	8.93348	8.79073	8.96481	0.14275	0.03133	−0.11142
13	C	5.66283	5.65255	5.67577	0.01028	0.01294	0.00266
14	O	8.93348	8.79073	8.96481	0.14275	0.03133	−0.11142
15	C	6.74004	6.49109	6.85198	0.24895	0.11194	−0.13701
18	C	6.74004	6.49109	6.85198	0.24895	0.11194	−0.13701

At this stage, no further reaction occurs because there is no reactive electrophilic site for self-condensation. Direct observation of the chemical descriptors presented in Table 2 indicates the reactivity of each relevant atom of **5**. This can be confirmed by the near-zero electrophilicity values for carbons 11 and 13. Further analysis of the results makes clear that C15 and C18 are the most nucleophilic sites of the molecule, with the lowest Δ*f*(*r*) values obtained.

**Scheme 4:**
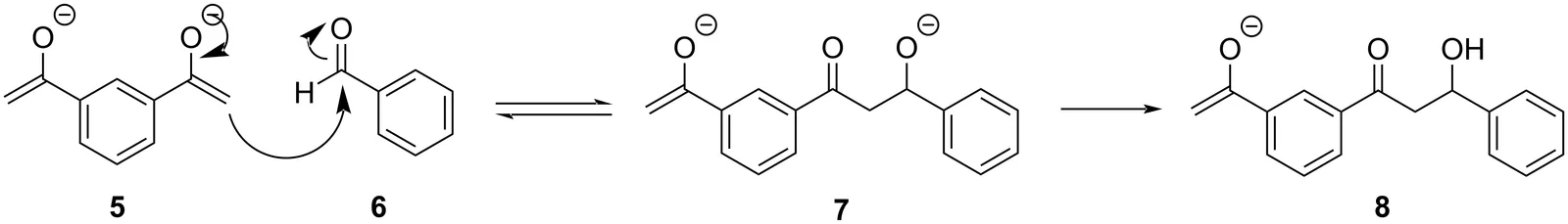
Addition step of the Claisen–Schmidt condensation mechanism in basic medium.

The next step is the addition of benzaldehyde **6**, a strong electrophile, to the solution. Benzaldehyde (used as a simplified model) does not contain the methoxy substituent (present in 4-methoxybenzaldehyde used in the performed experiments), which has a clear electronic effect, as it is an electron-donating group that increases the electron density of the aromatic ring and can influence the electrophilicity of the aldehyde carbonyl through resonance and inductive effects. However, it is not expected that this effect fundamentally changes the overall mechanistic picture of the Claisen–Schmidt condensation. The key elementary steps considered in our analysis – enolate formation, nucleophilic addition to the aldehyde, proton-transfer steps, and subsequent dehydration – remain chemically analogous. Thus, one enolate will attack the carbonyl group of the aldehyde, yielding an aldol (Scheme 4) [46].

**Figure 2:**
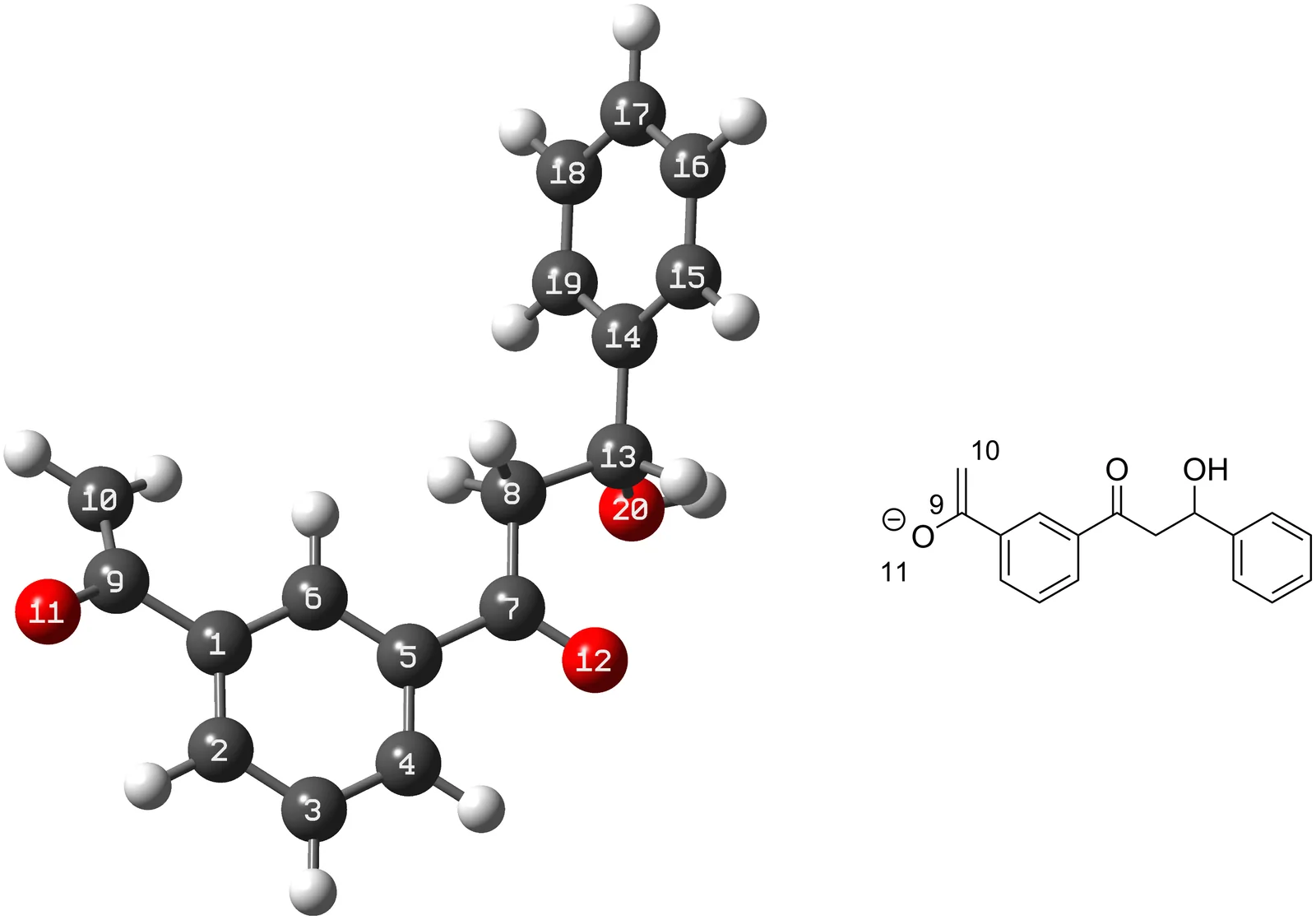
Optimized structure of compound **8** in methanol at the CAM-B3LYP/6-311++G(d,p) basis set level.

Because each side reacts separately, the new intermediate **8** has only one aldol group on one side and an enolate on the other (Figure 2).

**Scheme 5:**
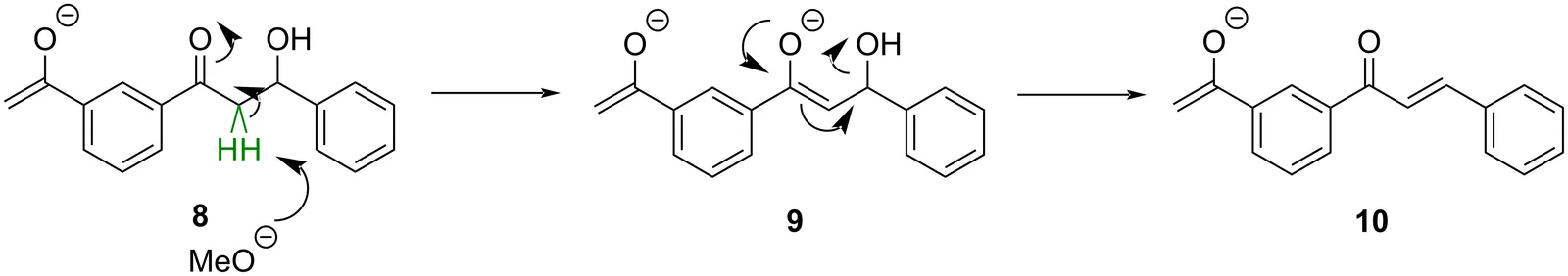
Dehydration step in the mechanism of the Claisen–Schmidt condensation.

The α-protons of the aldol moiety are acidic, and given the excess of base in solution, a new enolate will form. This enolate will stabilize through dehydration, in which the hydroxy group on the β-carbon leaves via a type-1 elimination mechanism (Scheme 5) [46].

Then the second condensation step can occur on the remaining enolate of the molecule **10**. The reaction mechanism is the same as previously mentioned, regardless of the nature of the substrates, as long as aromatic aldehydes and ketones are present [46]. However, the process becomes more complex in the presence of other reactive sites on the molecule.

**Figure 3:**
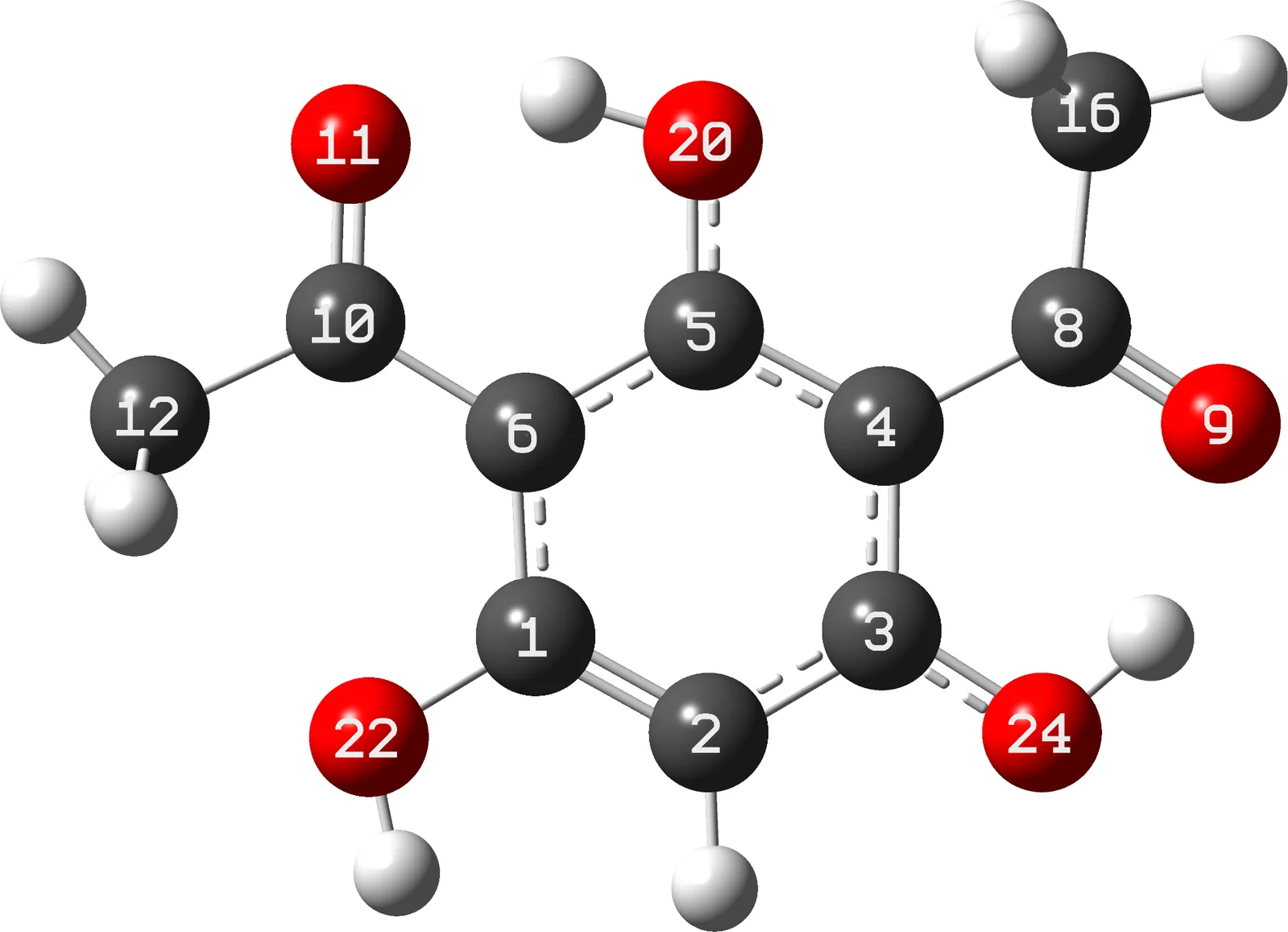
Optimized structure of **1** in methanol at the CAM-B3LYP/6-311++G(d,p) basis set level.

**Experimental reaction:** The same methodology was applied to the initial molecule, trihydroxy bisacetophenone **1**. The structure of this molecule was optimized using the CAM-B3LYP/6-311++g(d,p) basis set, and the resulting stable structure predicts two intramolecular hydrogen bonds and a hydroxy group with no internal bonding (Figure 3).

**Scheme 6:**
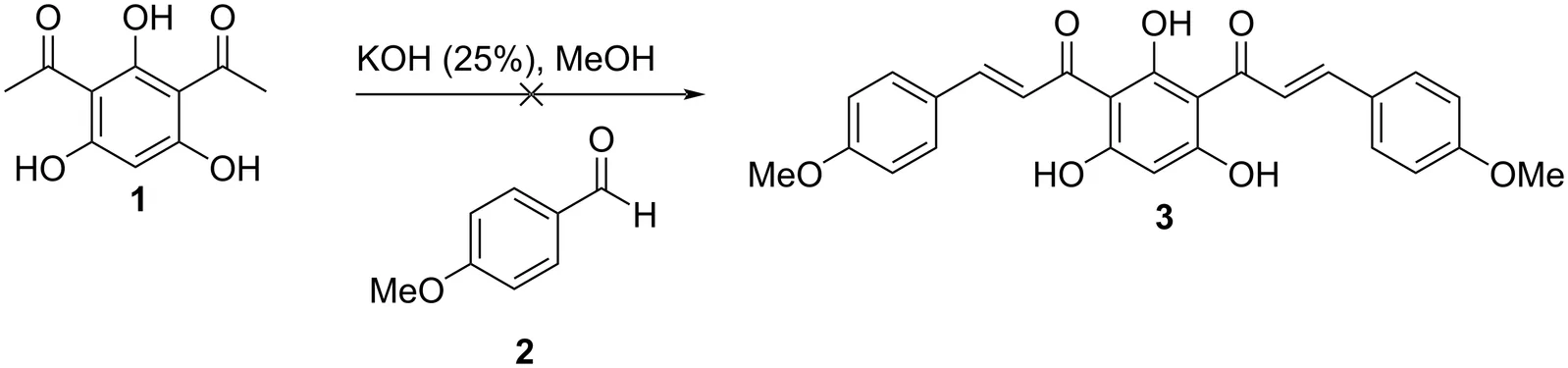
Attempted synthesis of bischalcone **3** in a basic medium.

This compound was expected to react with aldehyde **2** in a basic medium at a KOH concentration of about 1.8 M, always in excess relative to the amount of starting material (Scheme 6).

The main problem with this reaction is the presence of multiple hydroxy groups, all with nucleophilic potential.

**Scheme 7:**
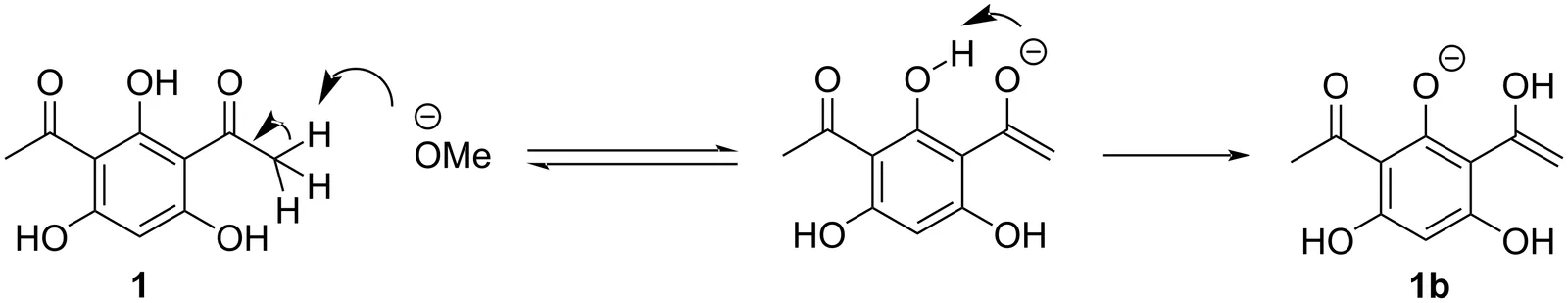
Single deprotonation of compound **1** followed by proton transfer.

**Figure 4:**
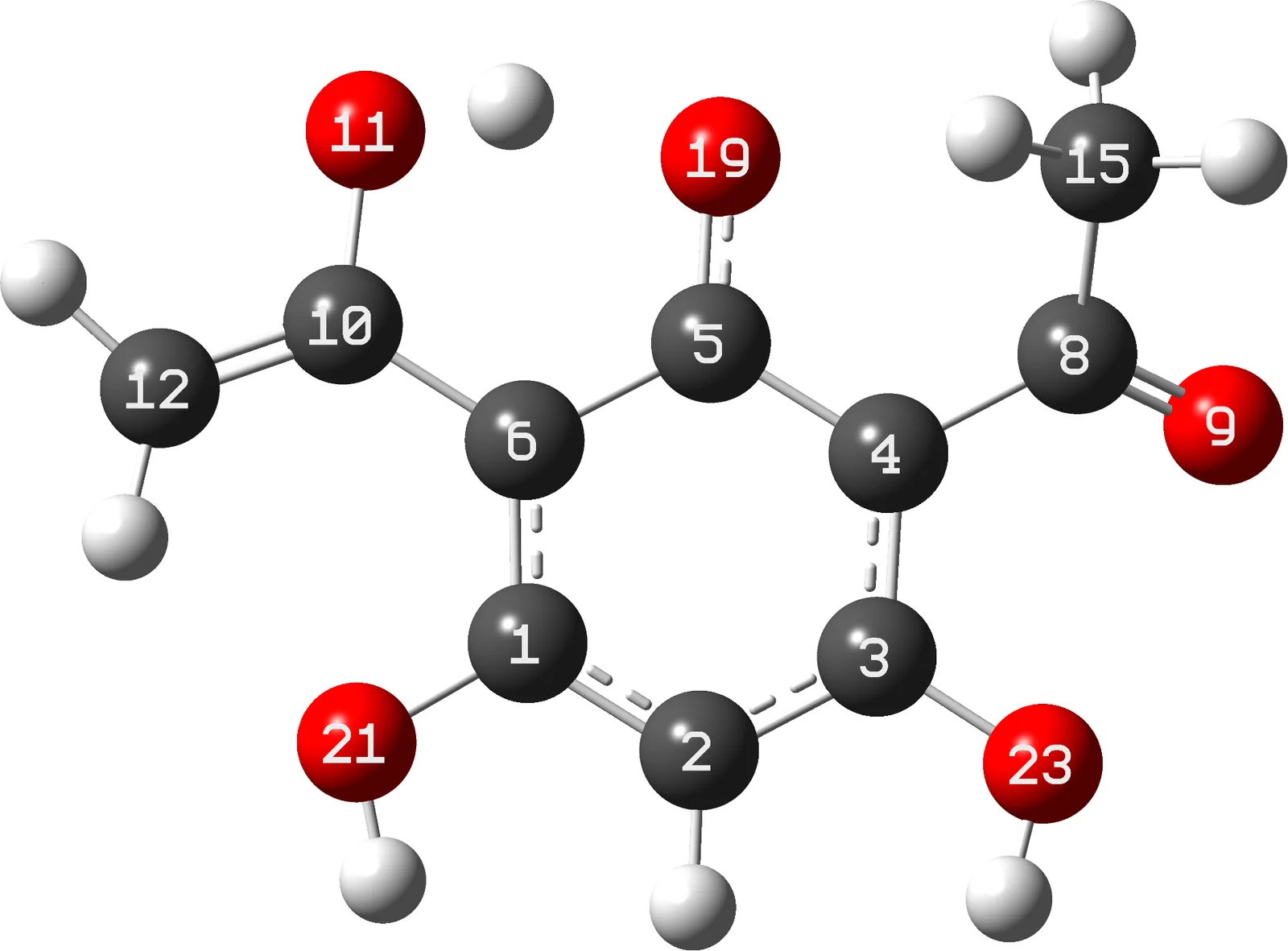
Optimized structure of **1b** in methanol, at the CAM-B3LYP/6-311++G(d,p) basis set level.

**Scheme 8:**
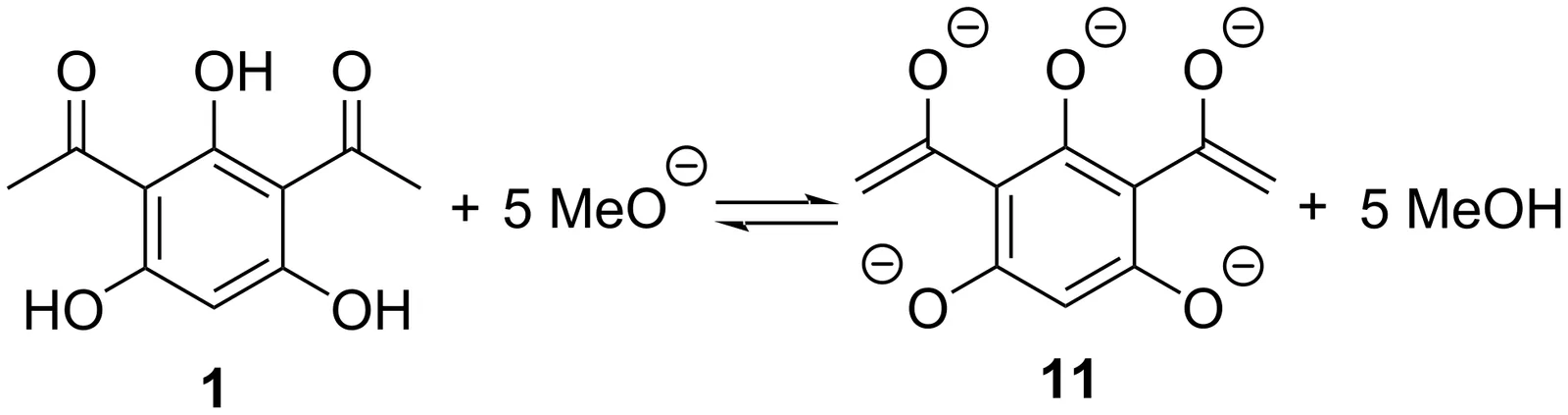
Enolate **11** formation step.

As mentioned above, the first step is the formation of enolates. However, in this case, several phenolates can be formed in addition to the enolates. Using DFT calculations with phenol as a reference acid, the predicted p*K*_a_ of the phenol protons is markedly different from that of the acidic α-protons. The first phenol deprotonation has a p*K*_a_ of 4.6, and the first α-protons have a p*K*_a_ of 19.1 (see the methodology explained in the Computational details section). Therefore, the base in solution will produce phenolic protons much sooner than the other protons (Scheme 7). Also, according to the optimized structure obtained by DFT (Figure 4), the removal of the α-proton will result in proton transfer between the adjacent phenol and the newly formed enolate, forming an enol intermediate **1b** with a phenolate moiety (Scheme 7). Therefore, phenolates will always be present before the formation of the intended enolates (Scheme 8).

**Figure 5:**
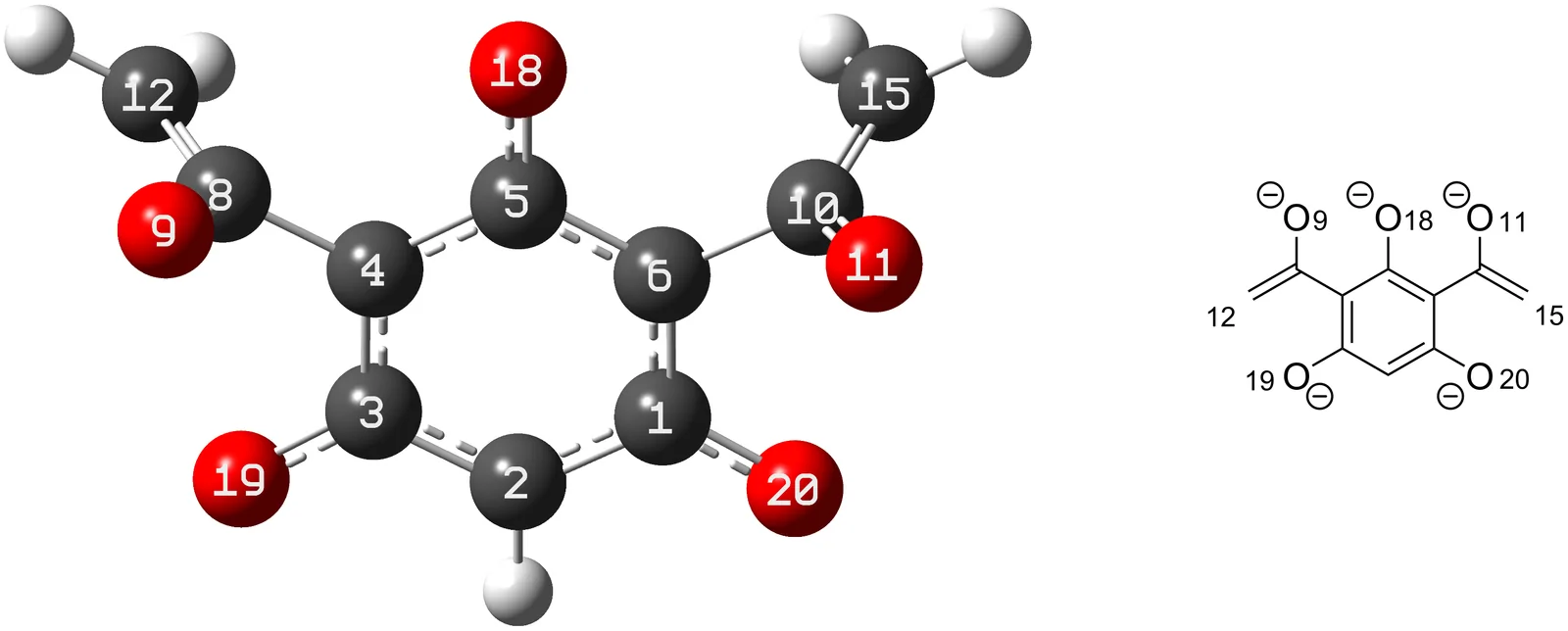
Optimized structure of **11** in methanol, at the CAM-B3LYP/6-311++G(d,p) basis set level.

These phenolates will compete for the nucleophilic attack and generate side reactions. They will also reduce the nucleophilic potential of the enolates (Figure 5 and Table 3).

**Table 3:** Condensed Fukui function values obtained for compound **11** from NBO charges in methanol solution (SMD solvent model) at the CAM-B3LYP/6-311++G(d,p) basis set level.

atom	type	*q*_k_(*N*)	*q*_k_(*N* – 1)	*q*_k_(*N* + 1)	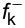	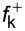	Δ*f*(*r*)

8	C	5.60965	5.64349	5.65392	−0.03384	0.04427	0.07811
9	O	8.99188	8.95713	8.99309	0.03475	0.00121	−0.03354
10	C	5.60963	5.64474	5.64641	−0.03511	0.03678	0.07189
11	O	8.99135	8.95591	8.99221	0.03544	0.00086	−0.03458
12	C	6.73489	6.7025	6.86832	0.03239	0.13343	0.10104
15	C	6.73511	6.70216	6.85162	0.03295	0.11651	0.08356
18	O	9.00739	8.96536	9.02434	0.04203	0.01695	−0.02508
19	O	9.02574	8.8631	9.02667	0.16264	0.00093	−0.16171
20	O	9.02494	8.86979	9.02541	0.15515	0.00047	−0.15468

As shown in Table 3, the α-carbons (C12 and C15) have a drastically different reactivity compared to the previous structure without hydroxy groups (Table 2). The previous values of nucleophilicity (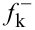) were around 0.25 for both carbons, higher than the 0.033 now obtained for C12 and C15, and the free hydroxy groups are more nucleophilic than the α-carbons, with Δ*f*(*r*) = −0.16 and −0.15 for both groups (O19 and O20).

With this information, we can confirm the hypothesis initially established for these basic reaction conditions. The nucleophilic potential of the phenolates is much higher than that of the enolates, so the most reactive sites in the molecules are not the ones needed to obtain the desired bischalcone. The only way to limit this issue would be to avoid the formation of the phenolates while forming the enolates. However, as seen by the p*K*_a_ values presented earlier, the deprotonation of the phenols will happen before the removal of the other protons. Also, an excess of base is required to favor the dehydration step [46], making this control over the amount of base non-viable.

### Acid-promoted condensation

**Scheme 9:**
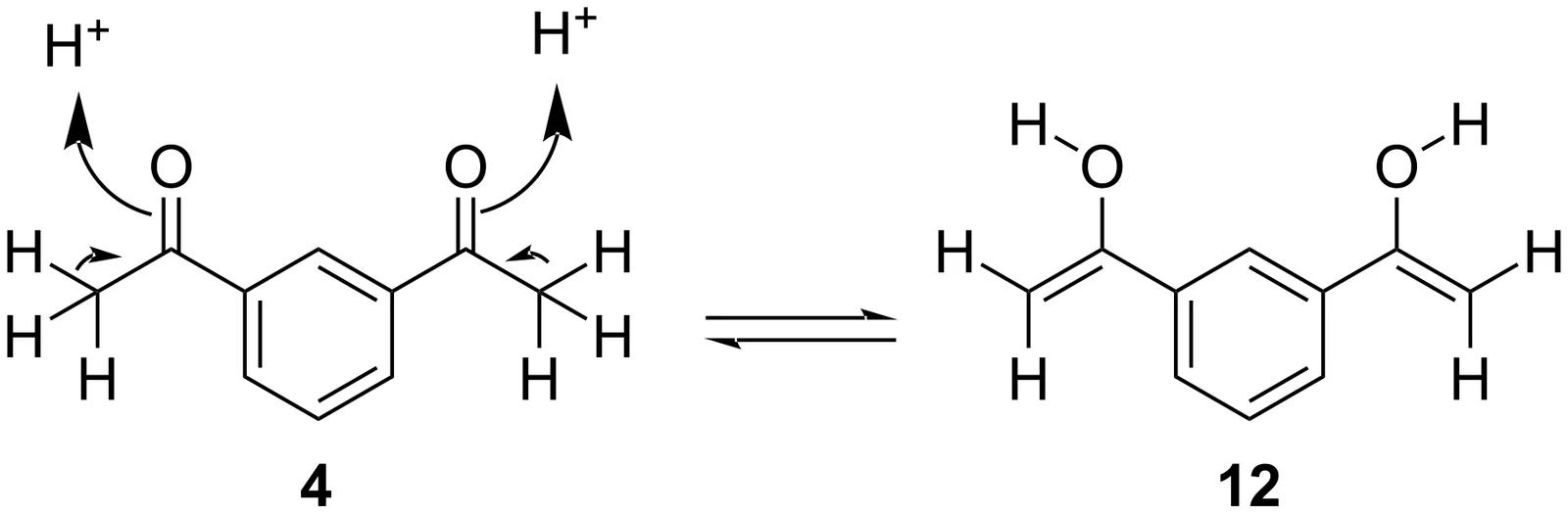
Enolization of **4** in an acidic medium.

**Model reaction*:*** A second alternative to attempt this reaction was the use of homogeneous acid conditions. The mechanism starts with protonation of both carbonyl groups if a Brønsted acid is used or complexation with a Lewis acid [47]. Then, the ketone moiety will reorganize into an enol, known as the keto–enol tautomerism (Scheme 9). For simplicity, the protonation model will be the one used.

**Figure 6:**
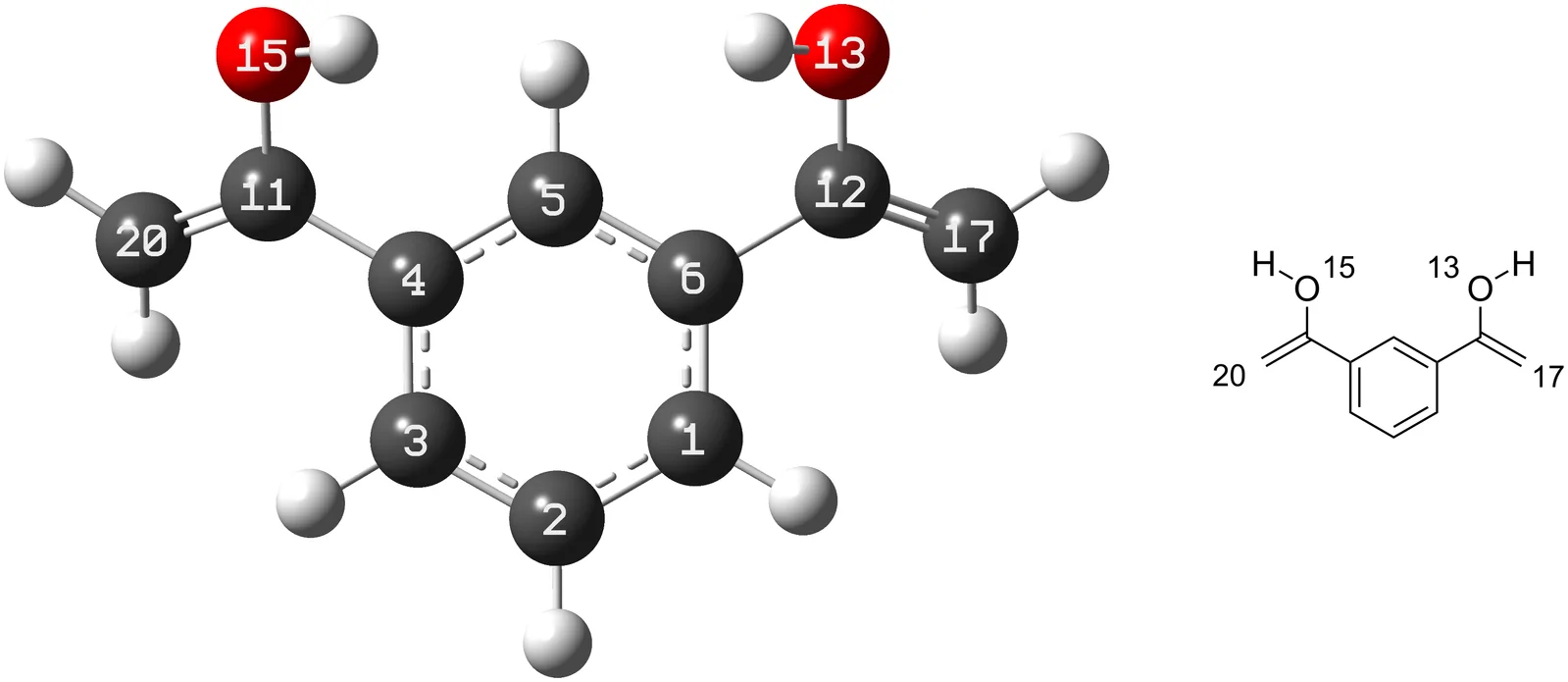
Optimized structure of **12** in methanol, at the CAM-B3LYP/6-311++G(d,p) basis set level.

The enol will act as a nucleophile, and the addition step will proceed, yielding an aldol. The reactivity of the atoms in this binucleophile (Figure 6) was calculated by natural population analysis of the optimized structure of the molecule, followed by the calculation of the respective Fukui functions (Table 4).

**Table 4:** Condensed Fukui function values obtained for compound **12** from NBO charges in methanol solution (SMD solvent model) at the CAM-B3LYP/6-311++G(d,p) basis set level.

atom	type	*q*_k_(*N*)	*q*_k_(*N* – 1)	*q*_k_(*N* + 1)	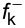	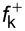	Δ*f*(*r*)

11	C	5.69162	5.67421	5.68649	0.01741	−0.00513	−0.02254
12	C	5.69162	5.67421	5.68649	0.01741	−0.00513	−0.02254
13	O	8.74219	8.69988	8.76053	0.04231	0.01834	−0.02397
15	O	8.74219	8.69988	8.76053	0.04231	0.01834	−0.02397
17	C	6.48971	6.30225	6.60866	0.18746	0.11895	−0.06851
20	C	6.48971	6.30225	6.60866	0.18746	0.11895	−0.06851

**Scheme 10:**
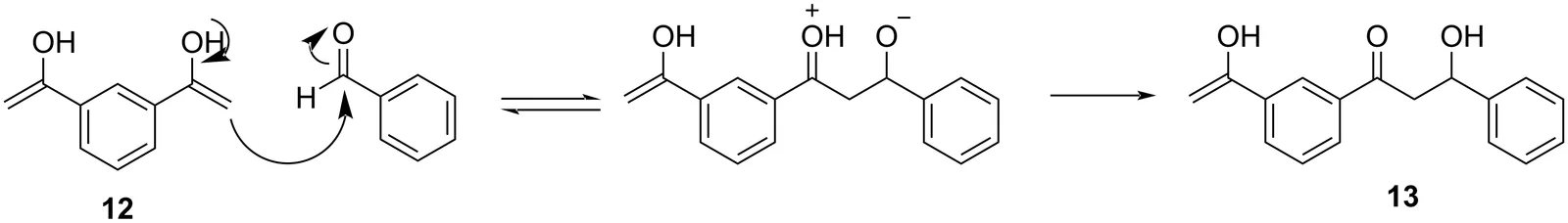
Addition step of the Claisen–Schmidt condensation mechanism in an acidic medium.

As shown in Table 4, the most reactive sites on the molecules are the two α-carbons of the enol groups (C17 and C20), in accordance with the reaction mechanism presented in Scheme 10. Note that the nucleophilicity of these carbons is lower than that of the same atoms of enolate **5** (Table 2). This also confirms the fact that enolates are more nucleophilic than their enol counterparts. The reaction can then proceed to the addition step, where the enol performs a nucleophilic attack on the aldehyde group (Scheme 10).

**Scheme 11:**
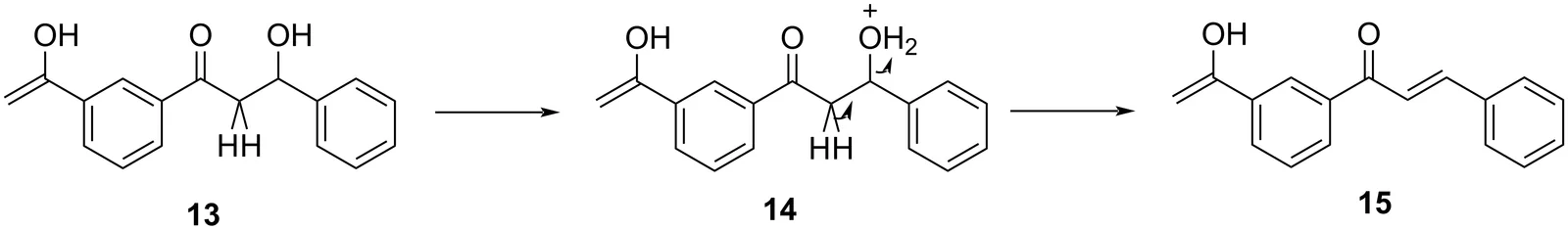
Dehydration step of the Claisen–Schmidt condensation mechanism in acidic medium.

The dehydration is genuinely acid-catalyzed as the acid is regenerated in the very last step. The hydroxy group of the aldol moiety will be protonated in acidic medium, yielding an OH_2_^+^, a good leaving group for the elimination step, and forming **15** (Scheme 11).

This reaction is possible experimentally with the regular chalcone derivatives that also possess other nucleophilic groups competing with the enol. Some authors have published methodologies for the synthesis of chalcones using acid in both catalytic and stoichiometric amounts for substrates containing some hydroxy groups [48–49].

**Scheme 12:**
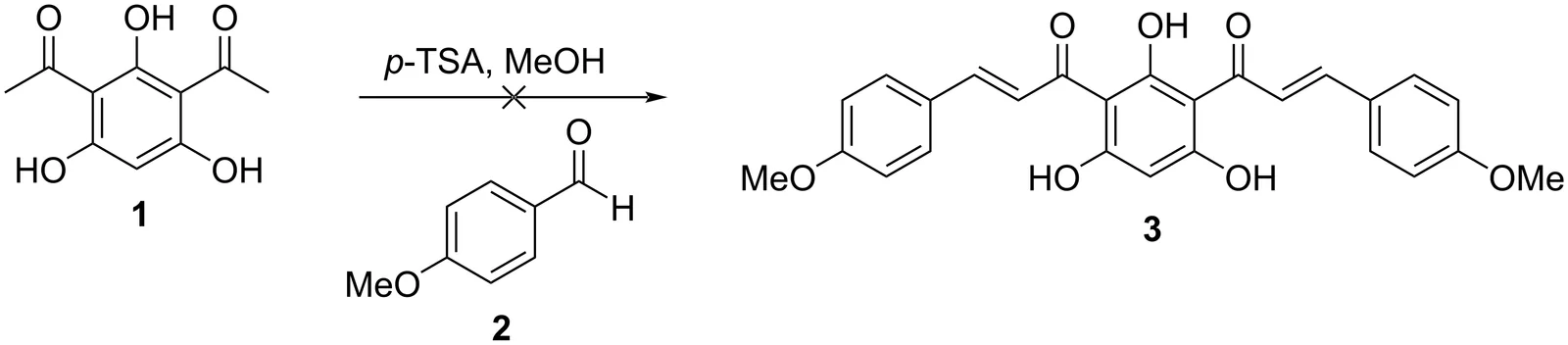
Attempted synthesis of bischalcone **3** in an acidic medium.

**Experimental reaction:** In this work, acid conditions were employed following two different methods: the reaction with a catalytic amount of *p*-TSA (Scheme 12), a protonic acid, and the reaction with boron trifluoride etherate, a Lewis acid. We explored the theoretical model of this reaction using protons as catalysts for simplification.

**Scheme 13:**
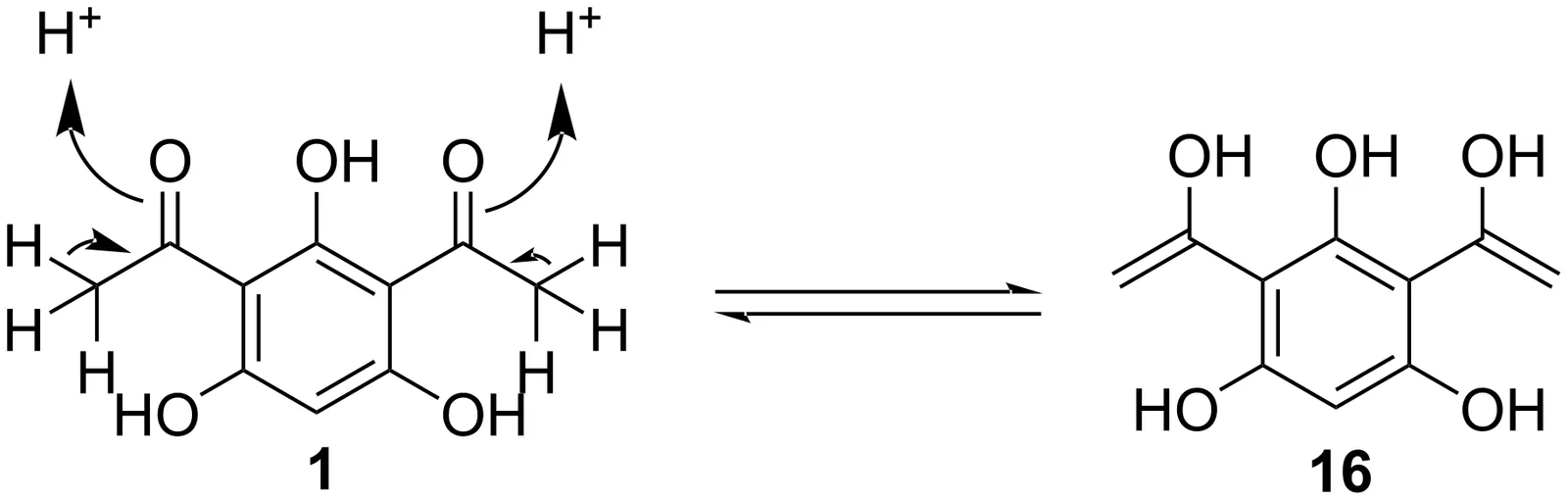
Enolization of **1** in an acidic medium.

In an acidic medium, two conjugated enol groups will be generated in solution after protonation of the carbonyl groups (Scheme 13).

**Figure 7:**
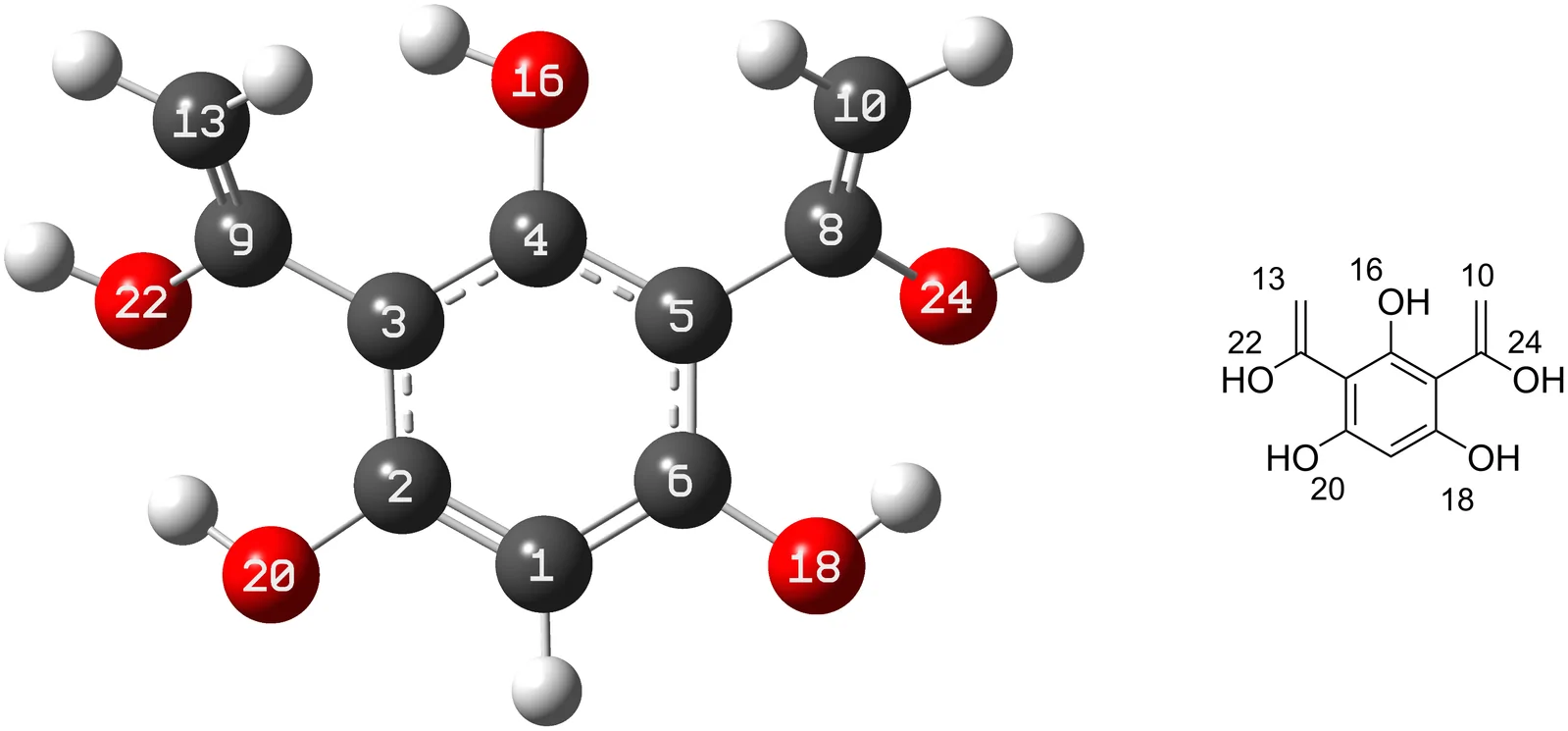
Optimized structure of **16** in methanol, at the CAM-B3LYP/6-311++G(d,p) basis set level.

In this molecule, there is competition between nucleophilic groups, specifically the hydroxy groups O6, O20, and O22, and two enol groups C10 and C13 (Figure 7, Table 5). In this case, the orientation of the O–H bond is relevant for the reactivity of the overall molecule, so two structures needed to be considered with two opposite orientations, since both are equally probable. The reactivity data presented in Table 5 are the average value of both orientations.

**Table 5:** Condensed Fukui function values obtained for compound **16** from NBO charges in methanol solution (SMD solvent model) at the CAM-B3LYP/6-311++G(d,p) basis set level.

atom	type	*q*_k_(*N*)	*q*_k_(*N* – 1)	*q*_k_(*N* + 1)	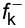	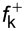	Δ*f*(*r*)

8	C	5.681745	5.6823	5.80112	−0.000555	0.119375	0.11993
9	C	5.6786	5.682305	5.80112	−0.003705	0.12252	0.126225
10	C	6.51393	6.36398	6.59324	0.14995	0.07931	−0.07064
13	C	6.51384	6.36398	6.59324	0.14986	0.0794	−0.07046
16	O	8.714525	8.67352	8.72692	0.041005	0.012395	−0.02861
18	O	8.72699	8.670265	8.73345	0.056725	0.00646	−0.050265
20	O	8.72947	8.670265	8.743545	0.059205	0.014075	−0.04513

The α-carbons C10 and C13 of the enol are the most nucleophilic sites in the molecule, even compared to the hydroxy groups (Table 5). This shows that the expected addition reaction is possible from a reactivity point of view, meaning that the failure to yield the intended bischalcone may not be related to the initial reactivity of the molecule. Therefore, the hypothesis initially proposed may not be valid for these acid reaction conditions. The problem could lie ahead, particularly with the stability of the bischalcone in these conditions. The formation of the intended bischalcone may be followed by a subsequent isomerization reaction, very common in 2’-hydroxychalcones. Successful examples of acid catalysis in polyhydroxylated chalcone and bischalcone synthesis usually lack any hydroxy groups in the 2-position of the A ring, to avoid this isomerization issue [1,21,50].

**Scheme 14:**
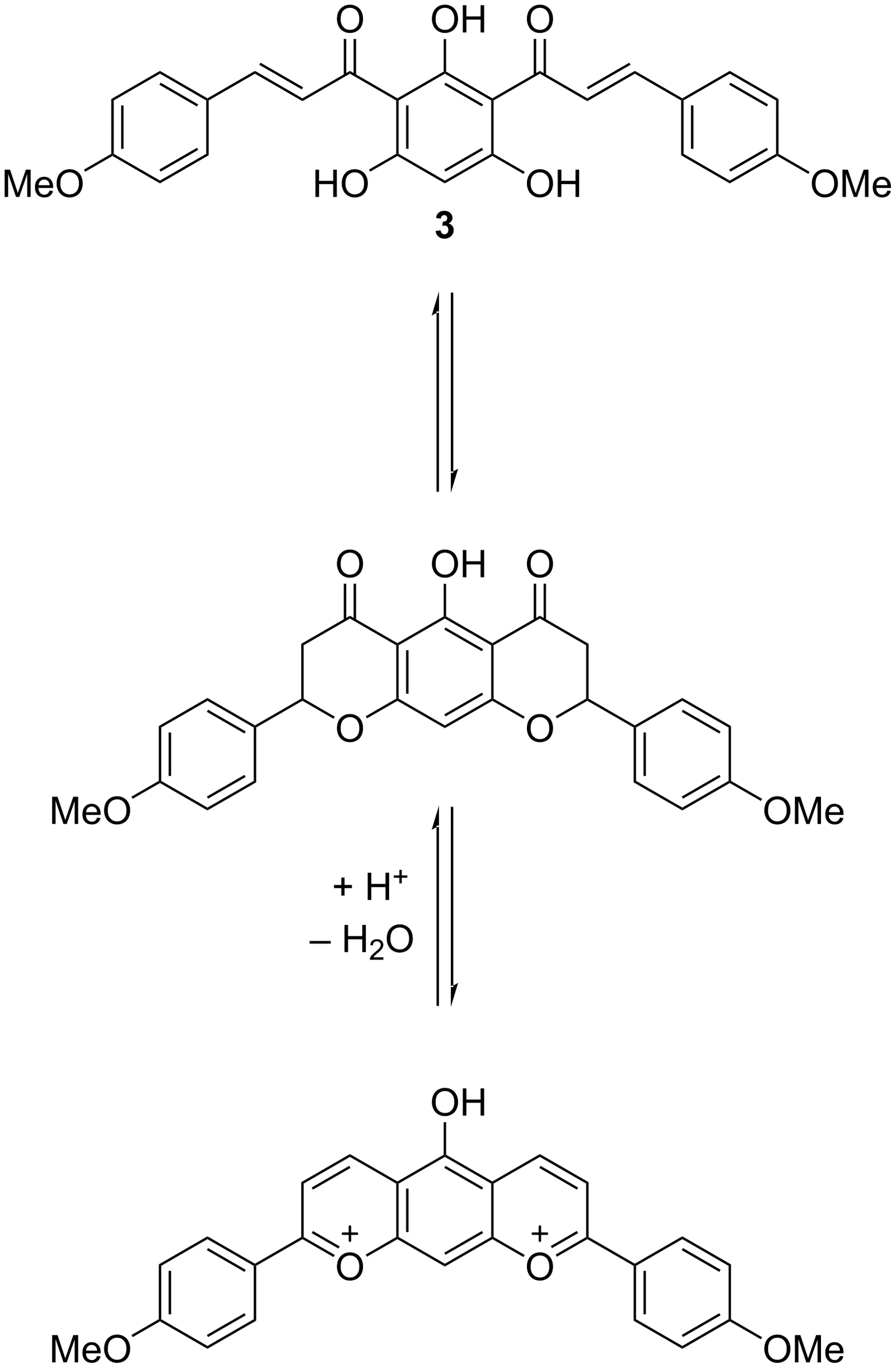
Cyclization of bischalcone **3** in an acid medium.

The isomerization/cyclization reaction previously mentioned can culminate in the formation of several flavonoid derivatives such as flavanones and deoxyanthocyanidins [51]. However, no such derivatives were isolated from the reaction crude because purification was unsuccessful due to extensive degradation. Since isomerization is catalyzed in an acidic medium, isolating the bischalcone before cyclization was not possible (Scheme 14). Therefore, the problem was not the Claisen–Schmidt condensation but keeping the bischalcone in solution and avoiding isomerization that may be promoted in acidic conditions.

Although these bischalcone isomerization/cyclization side reactions were experimentally inferred, without explicit calculations of the cyclization pathway and its activation barriers, the computational results do not independently establish that this pathway is responsible for the observed failure.

### Metal-assisted base-promoted condensation

The last main strategy employed to synthesize bischalcone **3** directly was the use of metal complexes. As initially stated, there are examples in the literature of successful direct cross-aldol condensation reactions between hydroxy ketones and aldehydes (base-sensitive compounds) catalyzed or assisted by alkali metal salts [16,52–53]. Calcium-based salts, such as CaO and Ca(OH)_2_, have been reported to be efficient catalysts for this reaction, as have lithium bases [17]. Both are used in equivalent quantities.

**Scheme 15:**
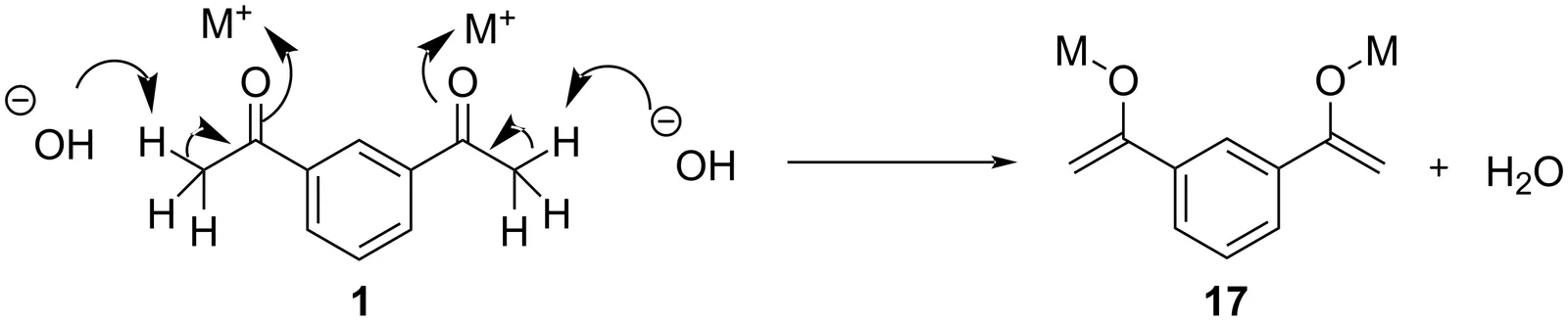
Enolate **17** and the metalation step in a basic medium.

**Model reaction*:*** The first step for this mechanism is the full deprotonation of all acidic groups. Both metal catalysts share the same mechanism; the basic anion will capture the acidic proton from the α-carbon, while the divalent cation coordinates with the resulting enolate **17** like a Lewis acid (Scheme 15).

**Scheme 16:**
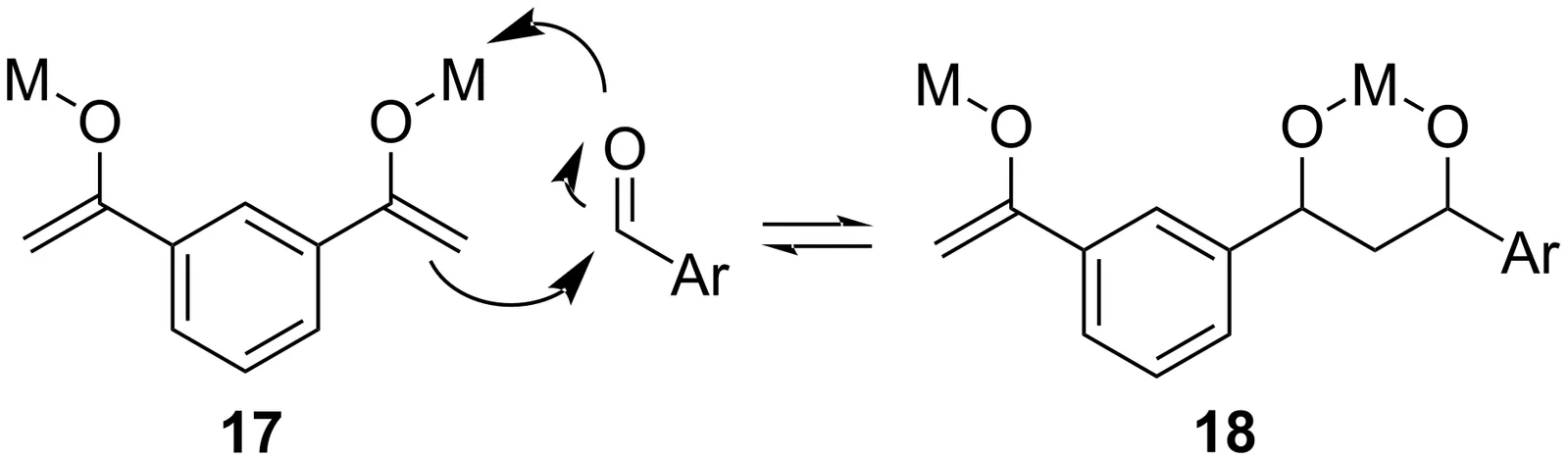
Addition step of the Claisen–Schmidt condensation mechanism in a basic medium with further enol stabilization by the metal cation.

The order of these two steps depends on the strength of the bonds formed. Some elements, such as boron, have a short bond length, about 1.4–1.6 Å, and therefore only bind to one oxygen of the molecule. However, calcium and lithium cations can complex with two or even four oxygen atoms [54]. The resulting aldol is further stabilized by coordination of the new hydroxy group with these divalent metals (Scheme 16).

**Scheme 17:**
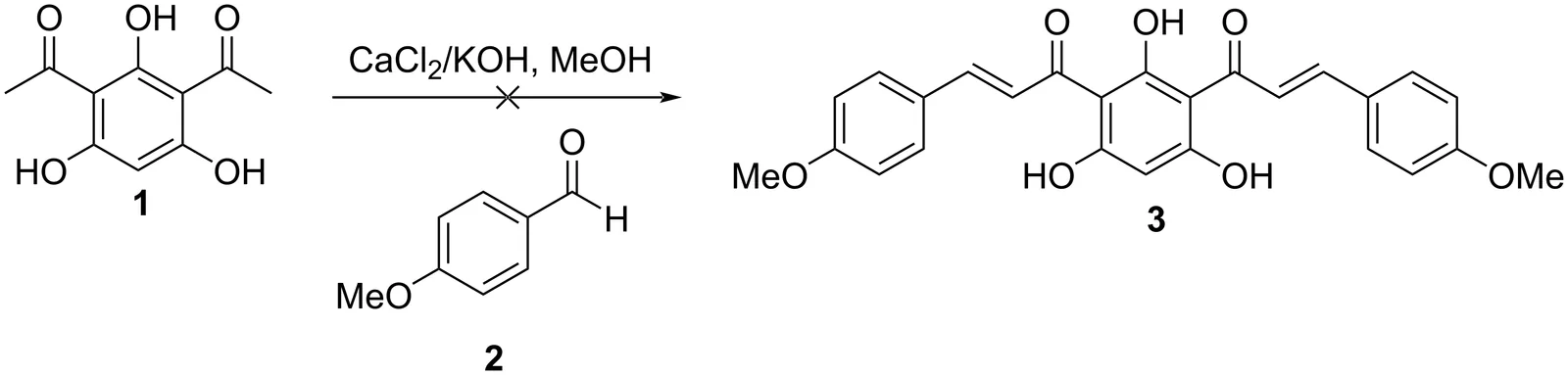
Attempted synthesis of bischalcone **3** in basic medium in the presence of calcium salts.

**Experimental reaction:** For the molecule under study, the reaction was initially attempted with two calcium-based bases, Ca(OH)_2_ and CaO (Scheme 17)

**Scheme 18:**
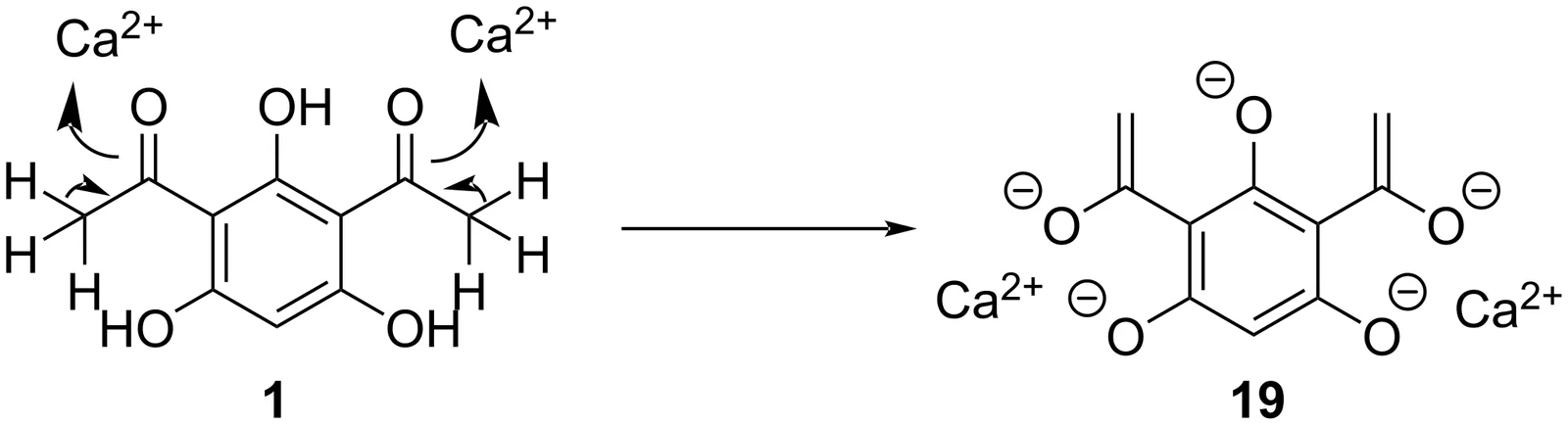
Enolate **19** formation step in the reaction with calcium hydroxide.

**Figure 8:**
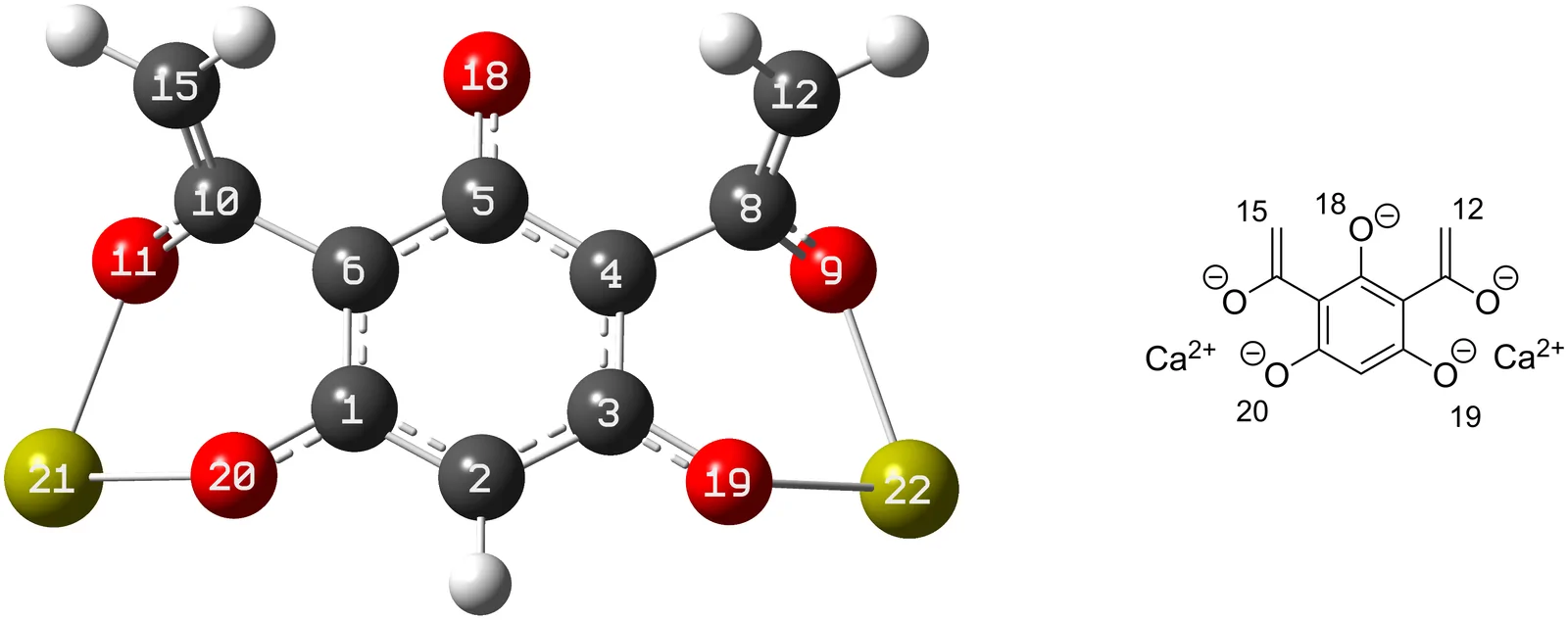
Optimized structure of **19** in methanol, at CAM-B3LYP/6-311++G(d,p) basis set level.

In the presence of hydroxy groups in the *o*-positions relative to the carbonyl group, the calcium cation coordinates with this oxygen atom, shifting and stabilizing the charge in this group, making it less reactive. The predicted complex is the one with two calcium cations coordinated to two carbonyl groups, as this is a known divalent metal (Scheme 18) [55]. During the structure optimization process, a third calcium atom was also positioned near oxygen 18 (Figure 8) for additional coordination. However, since this cation is divalent, it lacked an additional intramolecular position suitable for bonding at the right distance, leading to failure to predict a stable complex structure with three calcium cations.

The binding energy for the formation of complex **19** was −284.97 kJ·mol^−1^ (see the methodology described in the Computational details section and Supporting Information File 1). The negative value obtained indicates the spontaneous formation of this complex. The addition of extra calcium cations led to less stable molecules.

Geometrically, it would be impossible for the metal cation to coordinate with two phenolates at the same time because the distance between them is greater than the coordination radius. The distance of the calcium coordination bonds is about 2.2 Å, but the distance between the two phenolates is about 4.5 Å (Figure 8). Then, the reactivity of each atom of complex **19** was calculated and is presented in Table 6.

**Table 6:** Condensed Fukui function values obtained for compound **19** from NBO charges in methanol solution (SMD solvent model) at the CAM-B3LYP/6-311++G(d,p) basis set level.

atom	type	*q*_k_(*N*)	*q*_k_(*N* – 1)	*q*_k_(*N* + 1)	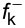	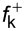	Δ*f*(*r*)

8	C	5.62519	5.6473	5.62441	−0.02211	−0.00078	0.02133
9	O	9.03312	9.01439	9.03067	0.01873	−0.00245	−0.02118
10	C	5.62519	5.6473	5.62441	−0.02211	−0.00078	0.02133
11	O	9.03313	9.01439	9.03067	0.01874	−0.00246	−0.02120
12	C	6.65813	6.61741	6.66483	0.04072	0.00670	−0.03402
15	C	6.65812	6.61741	6.66483	0.04071	0.00671	−0.03400
18	O	8.97593	8.70459	8.97782	0.27134	0.00189	−0.26945
19	O	9.03843	8.99615	9.03700	0.04228	−0.00143	−0.04371
20	O	9.03843	8.99615	9.03700	0.04228	−0.00143	−0.04371

As we can see in Table 6, the outstanding result is O18, the only oxygen that is not coordinated to a calcium atom. This oxygen has an exceptionally high nucleophilic potential, eclipsing the nucleophilicity of the α-carbons C12 and C15. Calcium acts to reduce the nucleophilicity of the other two phenolates, but the remaining one is much more active as a result, making it by far the most reactive site in the molecule.

**Scheme 19:**
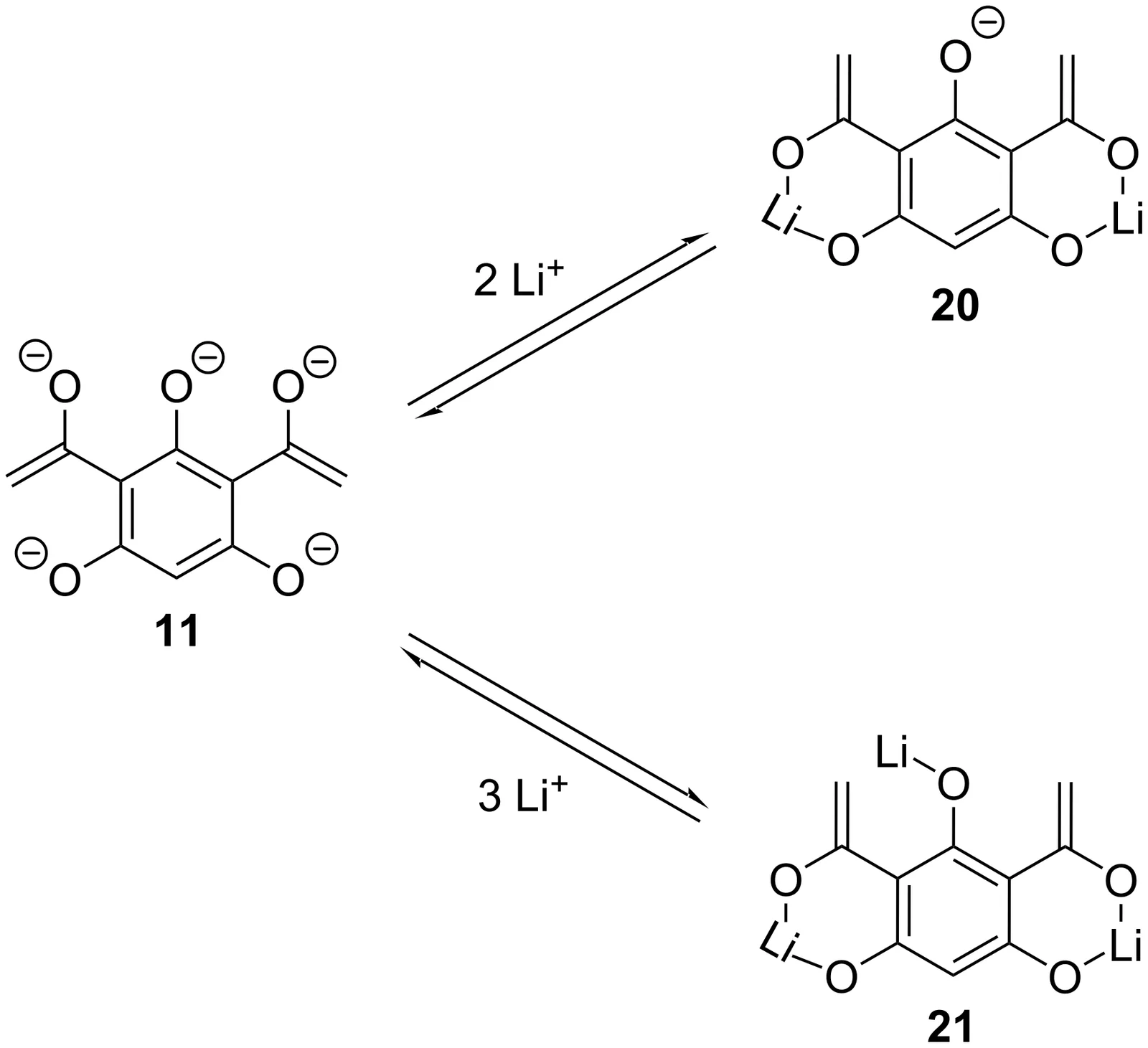
Formation of lithium cation enolates **20** and **21** in basic medium.

Lithium bases (LiOH and LiHMDS) have also been associated with the ability to catalyze cross-aldol condensation, exhibiting similar behavior to Ca^2+^ [56–57]. It has been known for many decades that lithium bases have a strong affinity for carbonyl and hydroxy groups [58]. The O–Li bond is strong, so the first step in enolate formation involves Li–O bond formation before the proton is removed from the α-position by the basic nitrogen atom of the amide [58–59]. Lithium cations have four coordination sites – two are possible within the same molecule, and those we do not show are occupied by either solvent molecules or another molecule of **11**. However, different combinations would be possible between lithium cations and **11**, since lithium can bind to phenolates, enolates or both (Scheme 19) [60].

Lithium enolates can exist as aggregates (dimers, tetramers, higher aggregates), and their aggregation state and structure are strongly dependent on the solvent and on the presence of coordinating species. In this study, the calculations were performed using the implicit SMD methanol model, which was selected to account for the overall solvent environment while keeping the computational treatment tractable. The main objective of the calculations was primarily to provide a qualitative mechanistic rationale and compare the relative feasibility of the proposed pathways rather than to quantitatively reproduce the aggregation equilibria of lithium enolates in solution.

**Figure 9:**
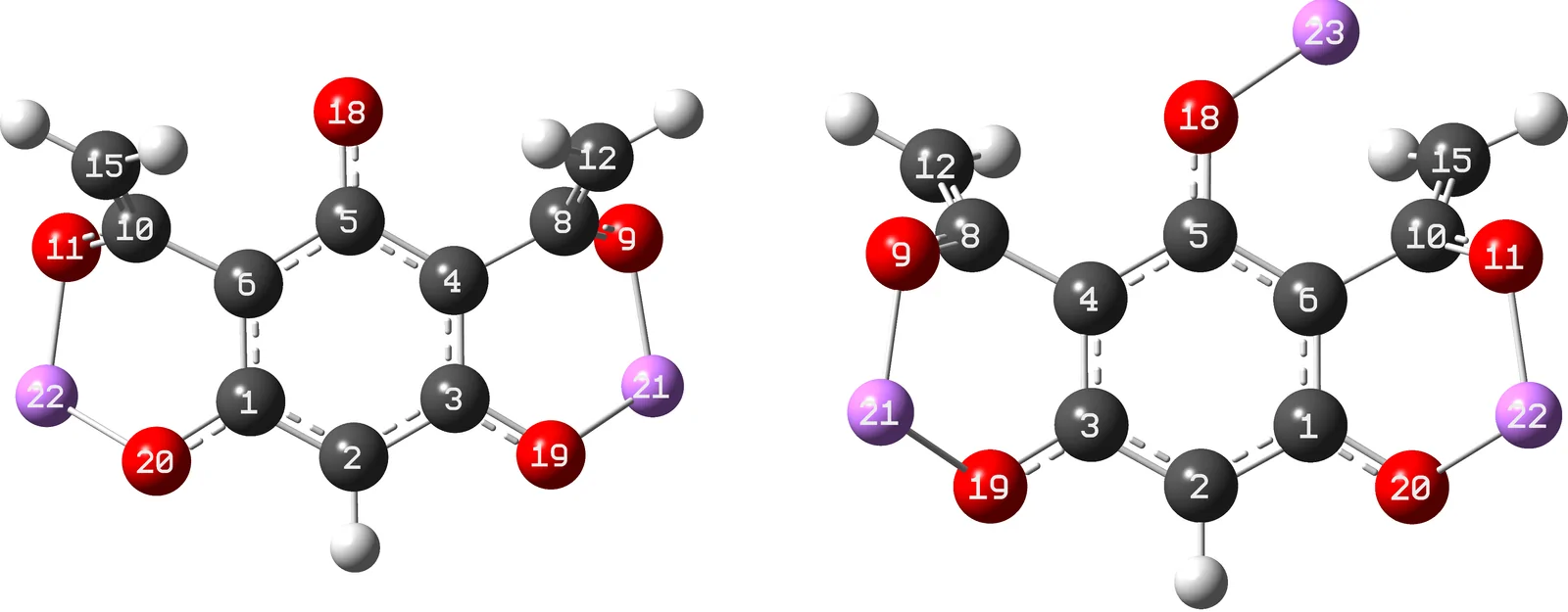
Optimized structures **20** and **21** in methanol, at the CAM-B3LYP/6-311++G(d,p) basis set level.

Two structures, **20** (binding energy = −399.327 kJ·mol^−1^) and **21** (binding energy = −559.956 kJ·mol^−1^) were selected as the most stable ones (Figure 9, Table 7 and Table 8), and we assumed two variations of **20** to account for the two possible orientations of the lithium atom that is single-bonded to the phenolate. We also assumed only one monomer with a variable number of coordinated lithium atoms, and we did not consider the possibilities of dimers and other conjugated structures with multiple molecules of **11**, as the focus is on the reactivity of a single molecule.

**Table 7:** Condensed Fukui function values obtained for compound **20** from NBO charges in methanol solution (SMD solvent model) at the CAM-B3LYP/6-311++G(d,p) basis set level.

atom	type	*q*_k_(*N*)	*q*_k_(*N* – 1)	*q*_k_(*N* + 1)	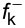	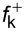	Δ*f*(*r*)

8	C	5.57789	5.59359	5.59536	−0.0157	0.01747	0.03317
9	O	8.95682	8.94535	8.96593	0.01147	0.00911	−0.00236
10	C	5.63242	5.68567	5.63266	−0.05325	0.00024	0.05349
11	O	9.00023	8.96187	9.00146	0.03836	0.00123	−0.03713
12	C	6.78532	6.76012	6.75517	0.0252	−0.03015	−0.05535
15	C	6.66056	6.52944	6.65925	0.13112	−0.00131	−0.13243
18	O	8.98734	8.90331	8.96271	0.08403	−0.02463	−0.10866
19	O	8.99747	8.87271	8.99994	0.12476	0.00247	−0.12229
20	O	8.99608	8.93713	8.99895	0.05895	0.00287	−0.05608

**Table 8:** Condensed Fukui function values obtained for compound **21** from NBO charges in methanol solution (SMD solvent model) at the CAM-B3LYP/6-311++G(d,p) basis set level.

atom	type	*q*_k_(*N*)	*q*_k_(*N* – 1)	*q*_k_(*N* + 1)	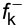	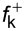	Δ*f*(*r*)

8	C	5.61361	5.65593	5.62926	−0.04232	0.01565	0.05797
9	O	9.01931	8.98731	9.00564	0.032	−0.01367	−0.04567
10	C	5.61361	5.65593	5.62926	−0.04232	0.01565	0.05797
11	O	9.01931	8.98731	9.00564	0.032	−0.01367	−0.04567
12	C	6.69332	6.62024	6.67049	0.07308	−0.02283	−0.09591
15	C	6.69332	6.62024	6.67049	0.07308	−0.02283	−0.09591
18	O	8.98516	8.71116	8.9794	0.274	−0.00576	−0.27976
19	O	9.02785	8.96622	9.0076	0.06163	−0.02025	−0.08188
20	O	9.02785	8.96622	9.0076	0.06163	−0.02025	−0.08188

A key challenge in this reaction arises from the differences between our initial complexes and the regular mechanism of action of lithium enolates. Typically, lithium cations coordinate with the two oxygen atoms of the resulting enol after the initial addition step, shifting the equilibrium towards more product formation. However, in this case, the lithium cations are already coordinated with two oxygens before the electrophile addition, reducing their ability to stabilize the resulting enol.

Lithium complexes enhanced stabilization and charge distribution (as shown in Table 8), reducing the nucleophilicity of the phenolates. However, they remain sufficiently nucleophilic to compete with the enolates, as evidenced by the similar reactivity values of the atoms involved. In structure **21**, the complex with the lowest binding energy, the α-carbons show higher reactivity than the stabilized lithium phenolates, but phenolates still compete substantially. This makes it difficult for the molecules to selectively follow the correct path, hindering the intended mechanism and leading to other side reactions.

## Conclusion

This study focused on the reactivity of the starting trihydroxy bisacetophenone **1** in the Claisen–Schmidt condensation with 4-methoxybenzaldehyde (**2**). Various experimental conditions (basic, acid, and metal-assisted basic conditions) were tested to synthesize trihydroxy bischalcone **3** directly, but all attempts failed, mostly due to the competitive reactivity of the hydroxy groups. Strategies to mitigate this problem, such as metal chelates or different catalysts, did not solve this issue. Other activation methods, such as ultrasonic waves, were also ineffective, mainly because they do not interfere with the core reaction mechanism. A hypothesis was proposed to explain experimental observations, and DFT calculations were employed as qualitative tools to interpret chemical reactivity. Experimental observations remain the primary basis for the proposed reaction behavior, whereas computational results are used as complementary evidence to rationalize the observed reactivity. Fukui-function and related computational analyses provided a qualitative electronic rationale for the experimentally observed behavior, rather than a quantitative prediction of reaction selectivity or definitive proof of the reaction pathway. Studies involving transition-state calculations, activation barriers, reaction-energy profiles, and explicit solvent molecules will be further necessary to distinguish competing pathways kinetically and elucidate the reaction mechanism.

Still, the results of this work suggest that, under strong basic conditions, full deprotonation of phenol groups led to competition between nucleophiles, with phenolates outperforming enolates, causing side reactions. Lithium and calcium complexation reduced the nucleophilicity of the two phenolates but failed to suppress the unwanted reactivity of the remaining phenolate. In acidic conditions, the enols exhibited superior nucleophilicity to the phenols, not validating our initial hypothesis. However, the acidic conditions may promote bischalcone isomerization, potentially leading to degradation or undesired cyclization and other byproduct formation.

Alternative approaches may yield different results. Exploring different solvents could reduce the nucleophilicity of the hydroxy groups or enhance catalyst solubility. Another less explored hypothesis was the role of heterogeneous catalysis, where surface interactions could alter reactivity differently from homogeneous systems. With acid catalysis, the main obstacle for this reaction might be isomerization/degradation of the bischalcone, and not the success of the Claisen–Schmidt condensation. Therefore, a future successful strategy must avoid the isomerization reaction that naturally occurs in an acid medium or assisted by light. To do so, different solvents could be tested, as well as lower temperatures, or weaker acids could be used to mitigate this effect. By avoiding the classical methods tested in this work and exploring different options, one can strive for a more favorable outcome for this reaction.

## Supporting Information

File 1 Experimental section and computational calculations.

## Data Availability

Data generated and analyzed during this study are available from the corresponding author upon reasonable request.
