# Supplementary material for: The challenges of direct Claisen–Schmidt condensation in the synthesis of polyhydroxylated bischalcones: experimental results supported by DFT calculations
Source: Beilstein J Org Chem. 2026 Oct 12;22:1392–409. doi: 10.3762/bjoc.22.114 (PMC13678684; doi:10.3762/bjoc.22.114)
Supplement: Supplementary file 1 [file Beilstein_J_Org_Chem-22-1392-s001.pdf]

BEILSTEIN JOURNAL OF ORGANIC CHEMISTRY

Supporting Information

for

The challenges of direct Claisen–Schmidt condensation in the synthesis of polyhydroxylated bischalcones: experimental results supported by DFT calculations

Rui Pereira, Mariela M. Nolasco, Vera L. M. Silva and Eduarda Fernandes

Beilstein J. Org. Chem. **2026,** *22,* 1392–1409. doi:10.3762/bjoc.22.114

Experimental section and computational calculations

License and Terms: This is a supporting information file under the terms of the Creative Commons Attribution License (https://creativecommons.org/licenses/by/4.0). Please note that the reuse, redistribution and reproduction in particular requires that the author(s) and source are credited and that individual graphics may be subject to special legal provisions.
The license is subject to the *Beilstein Journal of Organic Chemistry* terms and conditions: (https://www.beilstein-journals.org/bjoc/terms)

Table of contents

1. Experimental section S2

1.1. General S2

1.2. Synthesis S2

2. Computational calculations S4

2.1. G09 optimized geometries S4

2.1.1. Fukui functions S4

2.1.2. Thermodynamic cycles of deprotonation S9

1. Experimental section

1.1. General

Reagents and solvents were purchased as reagent-grade and used without further purification unless otherwise stated. Phloroglucinol was purchased from BDH Laboratory Reagents. The following reagents were purchased from Sigma Aldrich: 4-methoxybenzaldehyde, potassium hydroxide, *p*-toluenesulfonic acid (*p*-TSA), boron trifluoride diethyl etherate, calcium chloride, lithium bis(trimethylsilyl)amide, and lithium hydroxide. Reactions were controlled by thin-layer chromatography (TLC) using silica gel 60 HF254 plates and were observed under UV light at 254 and/or 365 nm. Column chromatography was performed using flash silica gel (40–60 µm, 60A). Melting points were determined with a Büchi melting point B-540 apparatus and are uncorrected.

1.2. Synthesis

The following protocols describe the synthetic procedures for each reaction listed in Table 1 of the Results and Discussion section.

i) KOH (4.0 g) was added to compound **1** (0.5 mmol, 100 mg) dissolved in methanol (40 mL). After stirring for 30 min at room temperature, 4-methoxybenzaldehyde (**2**, 2.1 equiv, 1.05 mmol, 143 mg) was added to the mixture. The mixture was stirred at room temperature for 24–48 h, poured into ice, and quenched with diluted hydrochloric acid to adjust the pH to 4. The mixture was extracted with DCM (3 × 20 mL). The organic layers were dried over anhydrous sodium sulfate, evaporated to dryness, and the residue was purified by column chromatography on silica gel using hexane/ethyl acetate (1:1) as eluent. Only starting benzaldehyde was recovered.

ii) A 1:2 mixture of $CaCl_2$/KOH (2.1 equiv, 1.05 mmol, 120 mg/60 mg) was added to compound **1** (0.5 mmol, 100 mg) dissolved in methanol (40 mL). After stirring for 30 min at room temperature, 4-methoxybenzaldehyde (**2**, 2.1 equiv, 1.05 mmol, 143 mg) was added to the mixture. The mixture was stirred at reflux for 24 h, then poured into ice and quenched with diluted hydrochloric acid to adjust the pH to 4. The mixture was extracted with DCM (3 × 20 mL). The organic layers were dried over anhydrous sodium sulfate and evaporated to dryness. Only benzaldehyde was recovered.

iii) LiHMDS (3 equiv, 1.5 mmol, 1.5 mL) was added to compound **1** (0.5 mmol, 100 mg) dissolved in dried THF (40 mL). After stirring for 30 min at room temperature, 4-methoxybenzaldehyde (**2**, 2.1 equiv, 1.05 mmol, 143 mg) was added to the mixture. The mixture was stirred at 50 °C for 24 h, then it was poured into ice and quenched with diluted hydrochloric acid to adjust the pH to 4. The mixture was extracted with DCM (3 × 20 mL). The organic layers were dried over anhydrous sodium sulfate and evaporated

to dryness. The residue was purified by silica gel column chromatography using hexane/ethyl acetate (1:1) as eluent, yet only starting benzaldehyde was recovered.

iv) LiOH (3 equiv, 1.5 mmol, 36 mg) was added to compound **1** (0.5 mmol, 100 mg) dissolved in methanol (40 mL). After stirring for 30 min at room temperature, 4-methoxybenzaldehyde (**2**, 2.1 equiv, 1.05 mmol, 143 mg) was added to the mixture. The mixture was stirred at room temperature for 24 h, then poured into ice and quenched with diluted hydrochloric acid to adjust the pH to 4. The mixture was extracted with DCM (3 × 20 mL). The organic layers were dried over anhydrous sodium sulfate and evaporated to dryness. The residue was purified by silica gel column chromatography using hexane/ethyl acetate (1:1) as eluent, yet only starting benzaldehyde was recovered.

v) CaO (2.1 equiv, 1.05 mmol, 60 mg) was added to compound **1** (0.5 mmol, 100 mg) dissolved in methanol (40 mL). After stirring for 30 min at room temperature, 4-methoxybenzaldehyde (**2**, 2.1 equiv, 1.05 mmol, 143 mg) was added to the mixture. The mixture was stirred at 50 °C for 24 h, then poured into ice and quenched with diluted hydrochloric acid to adjust the pH to 4. The mixture was extracted with DCM (3 × 20 mL). The organic layers were dried over anhydrous sodium sulfate and evaporated to dryness. All starting materials were recovered.

vi) and vii): $BF_3·Et_2O$ (6 equiv, 3.0 mmol, 0.4 mL) was added to compound **1** (0.5 mmol, 100 mg) dissolved in 1,4-dioxane (40 mL). After stirring for 30 min at room temperature, 4-methoxybenzaldehyde (**2**, 2.1 equiv, 1.05 mmol, 143 mg) was added to the mixture. The mixture was stirred at room temperature (vi) or heated at 80 °C (vii) for 24 h, poured into ice, and quenched with diluted potassium carbonate aqueous solution. The mixture was extracted with ethyl acetate (3 × 20 mL). The organic layers were dried over anhydrous sodium sulfate, evaporated to dryness, and the residue was purified by silica gel column chromatography with hexane/ethyl acetate (1:1) as eluent. Only starting benzaldehyde was recovered.

viii) Amberlyst15 (20 mg) was added to compound **1** (0.5 mmol, 100 mg) dissolved in methanol (40 mL). After stirring for 30 min at room temperature, 4-methoxybenzaldehyde (**2**, 2.1 equiv, 1.05 mmol, 143 mg) was added to the mixture. The mixture was stirred at room temperature for 24 h in an ultrasound bath. The mixture was extracted with DCM (3 × 20 mL). The organic layers were dried over anhydrous sodium sulfate and evaporated to dryness. All starting materials were recovered.

ix) To a stirring solution of compound **1** (0.5 mmol, 100 mg) and 4-methoxybenzaldehyde (**2**, 2.1 equiv, 1.05 mmol, 143 mg) in methanol (30 mL) was added *p*-TSA (0.1 equiv,

0.05 mmol, 10 mg). The resulting solution was stirred at room temperature for 24 h. Water was added, and the mixture was extracted with DCM (3 × 20 mL). The organic layers were dried over anhydrous sodium sulfate, evaporated to dryness, and the residue was purified by silica gel column chromatography with hexane/ethyl acetate (1:1) as eluent. Only starting benzaldehyde was recovered.

2. Computational calculations

2.1. G09 optimized geometries

2.1.1. Fukui functions

```
----------------------------------------------------------------------
 # opt=tight freq cam-b3lyp/6-311++g(d,p) scrf=(smd,solvent=methanol) pop=(full,nbo)
geom=connectivity
 ----------------------------------------------------------------------
```

Compound 5; E(RCAM-B3LYP) = -536.433289

C	-0.01044	1.20564	1.56090
C	0.00000	0.00000	2.24906
C	0.01044	-1.20564	1.56090
C	0.00051	-1.22115	0.16496
C	0.00000	0.00000	-0.50921
C	-0.00051	1.22115	0.16496
H	-0.03557	2.13672	2.11400
H	0.00000	0.00000	3.33395
H	0.03557	-2.13672	2.11400
H	0.00000	0.00000	-1.59163
C	0.00000	-2.50756	-0.62959
O	0.54785	-2.45558	-1.80704
C	0.00000	2.50756	-0.62959
O	-0.54785	2.45558	-1.80704
C	0.57998	3.61844	-0.09185
H	0.58799	4.54860	-0.65145
H	1.06174	3.60633	0.87656
C	-0.57998	-3.61844	-0.09185
H	-0.58799	-4.54860	-0.65145
H	-1.06174	-3.60633	0.87656

Compound 11; E(RCAM-B3LYP) = -760.643155

C	1.23925	1.39208	0.03232
C	0.00176	2.07087	0.03724
C	-1.23905	1.39680	0.02063
C	-1.23179	-0.02043	0.01228
C	-0.00229	-0.74124	-0.00458
C	1.22913	-0.02528	0.01970
H	0.00403	3.15908	0.04327
C	-2.53767	-0.77059	-0.05777
O	-2.97903	-1.09787	-1.25047

C	2.54201	-0.76419	-0.03752
O	3.09538	-0.90665	-1.21908
C	-3.20725	-1.07370	1.09312
H	-2.81487	-0.78775	2.06260
H	-4.15745	-1.60373	1.06533
C	3.10483	-1.24223	1.11141
H	2.61878	-1.11154	2.07177
H	4.04839	-1.78422	1.08890
O	-0.00738	-2.06538	-0.04372
O	-2.37131	2.09084	0.00830
O	2.37578	2.07850	0.04338

Compound 12; E(RCAM-B3LYP) = -537.388043

C	1.59363	-0.25117	1.20405
C	2.27305	-0.35840	0.00000
C	1.59363	-0.25117	-1.20405
C	0.21722	-0.02607	-1.21406
C	-0.46162	0.07598	0.00000
C	0.21722	-0.02607	1.21406
H	2.12868	-0.36245	2.13905
H	3.33971	-0.54989	0.00000
H	2.12868	-0.36245	-2.13905
H	-1.52616	0.28092	0.00000
C	-0.51606	0.11499	-2.49473
C	-0.51606	0.11499	2.49473
O	-1.81576	-0.32887	2.51024
H	-1.97053	-0.96725	1.80272
O	-1.81576	-0.32887	-2.51024
H	-1.97053	-0.96725	-1.80272
C	-0.01791	0.67870	3.59178
H	-0.60706	0.73340	4.49919
H	0.97267	1.11027	3.59118
C	-0.01791	0.67870	-3.59178
H	0.97267	1.11027	-3.59118
H	-0.60706	0.73340	-4.49919

Compound **16_1;** E(RCAM-B3LYP) = -763.091023

C	-0.01474	2.03795	-0.22383
C	-1.20609	1.33604	-0.15133
C	-1.23116	-0.06210	-0.00782
C	0.00302	-0.72367	0.06852
C	1.22559	-0.03960	-0.02108
C	1.18615	1.35824	-0.14816
H	-0.02565	3.11560	-0.32075
C	2.51761	-0.75392	0.02663
C	-2.51781	-0.78883	0.04321
C	2.82726	-1.87401	-0.62491

H	2.13567	-2.32226	-1.32529
H	3.79923	-2.33752	-0.49800
C	-2.81330	-1.94206	-0.54644
H	-3.80105	-2.37749	-0.44015
H	-2.07895	-2.46871	-1.13656
O	-0.02653	-2.06386	0.25668
H	0.87495	-2.40074	0.37992
O	2.31691	2.10610	-0.22621
H	3.07563	1.58941	0.09494
O	-2.34247	2.07219	-0.24267
H	-3.09874	1.53835	0.06184
O	-3.45610	-0.08700	0.77859
H	-4.32334	-0.50854	0.70296
O	3.41899	-0.10398	0.83799
H	4.29657	-0.50391	0.75730

Compound **16_2;** E(RCAM-B3LYP) = -763.091023

C	0.01475	2.03795	-0.22384
C	-1.18615	1.35824	-0.14816
C	-1.22560	-0.03959	-0.02108
C	-0.00303	-0.72367	0.06851
C	1.23116	-0.06210	-0.00783
C	1.20610	1.33604	-0.15134
H	0.02565	3.11561	-0.32075
C	2.51780	-0.78883	0.04321
C	-2.51761	-0.75392	0.02664
C	2.81330	-1.94207	-0.54641
H	2.07896	-2.46873	-1.13654
H	3.80105	-2.37750	-0.44011
C	-2.82728	-1.87398	-0.62494
H	-3.79924	-2.33749	-0.49804
H	-2.13569	-2.32220	-1.32535
O	0.02652	-2.06386	0.25666
H	-0.87496	-2.40073	0.37989
O	2.34247	2.07217	-0.24269
H	3.09874	1.53833	0.06184
O	-2.31690	2.10612	-0.22621
H	-3.07564	1.58946	0.09493
O	-3.41897	-0.10401	0.83804
H	-4.29656	-0.50393	0.75735
O	3.45609	-0.08699	0.77860
H	4.32333	-0.50853	0.70299

```
--------------------------------------------------------------------
# opt=(tight,verytight) freq cam-b3lyp/6-311++g(d,p) scrf=(smd,solvent=
methanol) pop=(full,nbo) scf=qc geom=connectivity iop(3/33=4)
--------------------------------------------------------------------
```

Compound **19;** E(RCAM-B3LYP) = -2115.827385

C	-1.22434	-0.73547	0.67844
C	0.00000	-1.37832	0.91932
C	1.22434	-0.73547	0.67844
C	1.23040	0.59365	0.19906
C	0.00000	1.27285	-0.07133
C	-1.23040	0.59365	0.19906
H	0.00000	-2.39652	1.29834
C	2.55984	1.23887	-0.05756
O	3.33697	0.65298	-0.96869
C	-2.55984	1.23887	-0.05756
O	-3.33697	0.65298	-0.96869
C	2.96492	2.33408	0.62925
H	2.32474	2.80232	1.36704
H	3.92394	2.79961	0.41724
C	-2.96492	2.33408	0.62925
H	-2.32474	2.80232	1.36704
H	-3.92394	2.79961	0.41725
O	-0.00000	2.47333	-0.59148
O	2.36737	-1.38775	0.91081
O	-2.36737	-1.38775	0.91081
Ca	-4.09044	-1.43446	-0.54218
Ca	4.09044	-1.43446	-0.54218

Compound **20**; E(RCAM-B3LYP) = -783.114604

C	-1.22481	1.27297	-0.23722
C	0.00000	1.94901	-0.34210
C	1.22481	1.27297	-0.23722
C	1.23178	-0.12920	-0.03739
C	0.00000	-0.84957	0.09563
C	-1.23178	-0.12920	-0.03739
H	0.00000	3.02348	-0.50335
C	2.56665	-0.80867	0.06487
O	3.36959	-0.40127	1.04963
C	-2.56665	-0.80867	0.06487
O	-3.36959	-0.40127	1.04963
C	2.95705	-1.75397	-0.82500
H	2.30064	-2.08008	-1.62265
H	3.91920	-2.24896	-0.72221
C	-2.95705	-1.75397	-0.82500
H	-2.30064	-2.08008	-1.62265
H	-3.91920	-2.24896	-0.72221
O	0.00000	-2.13169	0.36877
O	2.36528	1.96280	-0.33365
O	-2.36528	1.96280	-0.33365

Li	3.63353	1.35558	0.78047
Li	-3.63353	1.35558	0.78047

Compound **21_1**; E(RCAM-B3LYP) = -783.114688

C	-1.24511	1.28772	-0.25476
C	-0.00278	1.93060	-0.36138
C	1.20843	1.23845	-0.22334
C	1.17464	-0.16204	0.00119
C	-0.07486	-0.83698	0.12400
C	-1.28708	-0.11248	-0.02046
H	0.02131	3.00038	-0.54823
C	2.47628	-0.89571	0.13051
O	3.31976	-0.46782	1.04160
C	-2.63094	-0.77103	0.10521
O	-3.43940	-0.29233	1.04988
C	2.77973	-1.96105	-0.67863
H	2.15496	-2.19883	-1.53516
H	3.75152	-2.44075	-0.60558
C	-3.01276	-1.77164	-0.72493
H	-2.34857	-2.15167	-1.49140
H	-3.98182	-2.24923	-0.60780
O	-0.09558	-2.12925	0.41919
O	2.36089	1.89707	-0.31659
O	-2.36331	1.99730	-0.37920
Li	3.62225	1.29527	0.80436
Li	-3.68240	1.45548	0.70799
Li	1.30455	-3.18004	0.27238

Compound **21_1**; E(RCAM-B3LYP) = -783.114688

C	-0.30306	0.99487	-1.51242
C	-0.14770	1.91411	-0.46545
C	0.02125	1.50591	0.86620
C	0.06394	0.11947	1.16906
C	-0.10019	-0.83200	0.12826
C	-0.25926	-0.39221	-1.21799
H	-0.17305	2.97625	-0.69137
C	0.24869	-0.29476	2.60151
O	-0.65278	0.15245	3.47384
C	-0.42861	-1.36741	-2.34510
O	-1.50701	-1.24645	-3.08351
C	1.30395	-1.05373	2.98364
H	2.03203	-1.40761	2.26413
H	1.41762	-1.36226	4.01944
C	0.50822	-2.33656	-2.60055
H	1.47107	-2.32062	-2.09709
H	0.39100	-2.99261	-3.45825
O	-0.15585	-2.12695	0.40708

O	0.14102	2.42308	1.82309
O	-0.47959	1.43398	-2.75675
Li	-0.67773	1.94662	3.34868
Li	-1.65184	0.44850	-3.68917
Li	0.01838	-3.38596	-0.80561

2.1.2. Thermodynamic cycles of deprotonation

 # opt=tight freq cam-b3lyp/6-311++g(d,p) scrf=(smd,solvent=methanol) pop=(full,nbo) geom=connectivity
 ----

Compound **1**; E(RCAM-B3LYP) = -763.147987

C	-1.23309	1.35245	-0.00000
C	-0.06259	2.08097	-0.00000
C	1.16362	1.43503	-0.00000
C	1.26071	0.01565	-0.00000
C	0.04454	-0.70844	0.00000
C	-1.22796	-0.06731	0.00000
H	-0.09117	3.16372	-0.00001
C	2.58537	-0.60523	-0.00000
O	3.59533	0.12273	-0.00000
C	-2.43573	-0.88716	0.00000
O	-2.33847	-2.13030	0.00001
C	-3.81806	-0.31550	0.00001
H	-4.52616	-1.14283	0.00001
H	-3.97941	0.31119	0.87774
H	-3.97942	0.31119	-0.87773
C	2.80724	-2.08443	0.00000
H	3.88118	-2.26573	0.00000
H	2.35207	-2.54643	0.87687
H	2.35207	-2.54644	-0.87687
O	0.10399	-2.02863	0.00000
H	-0.84813	-2.36301	0.00001
O	-2.41224	1.98969	-0.00000
H	-2.28198	2.94862	-0.00000
O	2.24895	2.20260	-0.00001
H	3.03617	1.58471	-0.00000

Compound **1** – H (α carbon); E(RCAM-B3LYP) = -762.659636

C	-1.22140	1.37456	-0.03867
C	-0.02498	2.09001	-0.04923
C	1.17529	1.41786	-0.02003
C	1.22906	-0.00505	0.00985
C	-0.01799	-0.73445	0.00651
C	-1.27066	-0.02163	-0.00215

H	-0.03612	3.17297	-0.07806
C	2.52207	-0.65154	0.03902
O	3.57212	0.03897	0.03427
C	-2.54890	-0.78214	0.02071
O	-2.44148	-2.10292	-0.32190
C	-3.77339	-0.33096	0.33331
H	-3.96922	0.69193	0.60115
H	-4.59981	-1.03256	0.31306
C	2.70920	-2.13791	0.07835
H	3.77930	-2.34354	0.10944
H	2.21943	-2.57276	0.94995
H	2.26444	-2.61083	-0.79781
O	-0.01359	-2.01947	0.01096
H	-1.45677	-2.32175	-0.24737
O	-2.38088	2.07304	-0.07440
H	-2.19530	3.02034	-0.11916
O	2.29731	2.15777	-0.02324
H	3.05632	1.50458	-0.00279

Compound **1** - H (OH); E(RCAM-B3LYP) = -762.690702

C	-1.19597	-1.39995	-0.00003
C	-0.00000	-2.09635	-0.00000
C	1.19597	-1.39995	0.00001
C	1.25203	0.02385	-0.00000
C	0.00001	0.77403	-0.00003
C	-1.25202	0.02385	-0.00002
H	-0.00001	-3.17818	0.00001
C	2.54199	0.66240	-0.00000
O	3.59609	-0.03023	0.00007
C	-2.54199	0.66240	0.00002
O	-3.59608	-0.03024	-0.00001
C	-2.73495	2.14908	0.00011
H	-3.80629	2.35130	0.00022
H	-2.27027	2.60557	0.87455
H	-2.27044	2.60563	-0.87438
C	2.73493	2.14908	-0.00007
H	3.80627	2.35132	-0.00012
H	2.27027	2.60556	-0.87452
H	2.27037	2.60561	0.87441
O	0.00002	2.02728	-0.00007
O	-2.31616	-2.12766	-0.00006
H	-3.07587	-1.46470	-0.00006
O	2.31616	-2.12766	0.00005
H	3.07585	-1.46468	0.00007

Phenol; E(RCAM-B3LYP) = -307.409453

C	-1.84852	0.03319	0.00001

C	-1.17210	-1.18220	0.00000
C	0.21472	-1.21933	-0.00000
C	0.93170	-0.02813	-0.00000
C	0.26817	1.19339	-0.00000
C	-1.12092	1.21670	0.00000
H	-2.93172	0.05665	0.00001
H	-1.72790	-2.11306	0.00000
H	0.75094	-2.16122	-0.00000
H	0.83988	2.11552	-0.00000
H	-1.63505	2.17115	0.00001
O	2.29684	-0.11470	-0.00001
H	2.69091	0.76683	-0.00001

Phenolate: E(RCAM-B3LYP) = -306.865941

C	0.00000	0.00000	-1.81197
C	0.00000	1.19584	-1.09630
C	0.00000	1.20285	0.28994
C	0.00000	0.00000	1.04423
C	0.00000	-1.20285	0.28994
C	0.00000	-1.19584	-1.09630
H	0.00000	0.00000	-2.89571
H	0.00000	2.14190	-1.62983
H	0.00000	2.14468	0.83084
H	0.00000	-2.14468	0.83084
H	0.00000	-2.14190	-1.62983
O	0.00000	0.00000	2.34706

----

opt=tight freq cam-b3lyp/6-311++g(d,p) pop=(full,nbo) geom=connectivity

----

Compound **1**; E(RCAM-B3LYP) = -763.127643

C	-1.22734	1.35168	0.00001
C	-0.06233	2.08057	0.00000
C	1.17230	1.43467	-0.00001
C	1.25905	0.01702	-0.00000
C	0.04487	-0.70792	0.00001
C	-1.22773	-0.06452	0.00001
H	-0.07418	3.16371	0.00000
C	2.59106	-0.60252	-0.00001
O	3.59388	0.12067	-0.00002
C	-2.43524	-0.89125	0.00000
O	-2.33749	-2.12647	-0.00006
C	-3.82319	-0.31643	0.00001
H	-4.52277	-1.14947	-0.00002
H	-3.98511	0.31408	0.87419
H	-3.98509	0.31414	-0.87413
C	2.80437	-2.08740	0.00001
H	3.87755	-2.26652	0.00000

H	2.33832	-2.54690	0.87169
H	2.33831	-2.54692	-0.87166
O	0.10359	-2.02544	0.00003
H	-0.85031	-2.35767	0.00008
O	-2.41689	1.99188	0.00001
H	-2.27161	2.94338	0.00001
O	2.24748	2.19800	-0.00001
H	3.03536	1.57965	-0.00001

Compound **1** – H (α carbon); E(RCAM-B3LYP) = -762.576299

C	-1.21439	1.36362	-0.03725
C	-0.01486	2.08425	-0.04005
C	1.19036	1.41962	-0.01355
C	1.23259	-0.00251	0.00811
C	-0.01691	-0.74214	0.00155
C	-1.27911	-0.02291	-0.00634
H	-0.01763	3.16857	-0.06105
C	2.52071	-0.64977	0.03207
O	3.58156	0.01919	0.03439
C	-2.56077	-0.78561	0.01922
O	-2.45764	-2.11015	-0.25120
C	-3.78663	-0.29969	0.28091
H	-3.96957	0.73978	0.47974
H	-4.61494	-0.99761	0.27529
C	2.67682	-2.14593	0.05784
H	3.74448	-2.36600	0.08638
H	2.15973	-2.57514	0.91585
H	2.20287	-2.59788	-0.81346
O	-0.00030	-2.01245	0.00089
H	-1.48131	-2.33107	-0.19704
O	-2.36779	2.09591	-0.07230
H	-2.12348	3.02401	-0.11298
O	2.31324	2.14992	-0.01119
H	3.06020	1.48244	0.00740

Compound **1** - H (OH); E(RCAM-B3LYP) = -762.615661

C	1.19901	-1.40702	0.00000
C	-0.00000	-2.10239	0.00000
C	-1.19901	-1.40701	-0.00001
C	-1.25189	0.01786	-0.00000
C	0.00000	0.77844	-0.00001
C	1.25189	0.01785	0.00000
H	-0.00001	-3.18310	-0.00000
C	-2.53800	0.65375	0.00000
O	-3.60236	-0.01705	-0.00000
C	2.53801	0.65375	0.00000
O	3.60236	-0.01704	0.00001

C	2.69999	2.15070	-0.00001
H	3.76897	2.36673	-0.00001
H	2.20745	2.59401	-0.86538
H	2.20747	2.59401	0.86537
C	-2.69999	2.15070	0.00002
H	-3.76897	2.36673	0.00004
H	-2.20745	2.59401	0.86539
H	-2.20748	2.59403	-0.86536
O	0.00001	2.02420	-0.00002
O	2.32176	-2.12431	0.00001
H	3.07013	-1.44915	0.00002
O	-2.32177	-2.12429	-0.00001
H	-3.07015	-1.44913	-0.00000

Phenol; E(RCAM-B3LYP) = -307.395317

C	1.84736	0.02837	-0.00000
C	1.16566	-1.18346	-0.00000
C	-0.21911	-1.21683	0.00000
C	-0.93364	-0.02482	0.00000
C	-0.26313	1.19211	0.00000
C	1.12549	1.21257	-0.00000
H	2.92979	0.04766	-0.00001
H	1.71868	-2.11529	-0.00000
H	-0.76300	-2.15290	0.00000
H	-0.82412	2.12140	-0.00000
H	1.64228	2.16488	-0.00000
O	-2.29491	-0.11042	0.00000
H	-2.68017	0.76998	0.00002

Phenolate; E(RCAM-B3LYP) = -306.830452

C	1.82051	0.00000	0.00000
C	1.09482	-1.19435	0.00000
C	-0.28672	-1.20654	0.00000
C	-1.07197	0.00000	0.00000
C	-0.28672	1.20654	-0.00000
C	1.09482	1.19435	-0.00000
H	2.90493	0.00000	0.00000
H	1.63012	-2.14237	0.00000
H	-0.83173	-2.14613	0.00000
H	-0.83173	2.14613	-0.00000
H	1.63012	2.14237	-0.00000
O	-2.33628	0.00000	0.00000

Metals

Ca^{2+}: E(RCAM-B3LYP) = -677.522648

Ca -3.17062 -3.70868 -1.29272

Li^{+}: E(RCAM-B3LYP) = -7.404105

Li -3.69854 -4.80485 -1.29272
